# Economic Analysis of the Impact of Overseas and Domestic Treatment and Screening Options for Intestinal Helminth Infection among US-Bound Refugees from Asia

**DOI:** 10.1371/journal.pntd.0004910

**Published:** 2016-08-10

**Authors:** Brian Maskery, Margaret S. Coleman, Michelle Weinberg, Weigong Zhou, Lisa Rotz, Alexander Klosovsky, Paul T. Cantey, LeAnne M. Fox, Martin S. Cetron, William M. Stauffer

**Affiliations:** 1 Division of Global Migration and Quarantine, Centers for Disease Control and Prevention, Atlanta, Georgia, United States of America; 2 International Organization for Migration, Washington, D.C., United States of America; 3 Division of Parasitic Diseases and Malaria, Centers for Disease Control and Prevention, Atlanta, Georgia, United States of America; 4 Division of Infectious Diseases and International Medicine, University of Minnesota, Minneapolis, Minnesota, United States of America; McGill University Health Centre, CANADA

## Abstract

**Background:**

Many U.S.-bound refugees travel from countries where intestinal parasites (hookworm, *Trichuris trichuria*, *Ascaris lumbricoides*, and *Strongyloides stercoralis*) are endemic. These infections are rare in the United States and may be underdiagnosed or misdiagnosed, leading to potentially serious consequences. This evaluation examined the costs and benefits of combinations of overseas presumptive treatment of parasitic diseases vs. domestic screening/treating vs. no program.

**Methods:**

An economic decision tree model terminating in Markov processes was developed to estimate the cost and health impacts of four interventions on an annual cohort of 27,700 U.S.-bound Asian refugees: 1) “No Program,” 2) U.S. “Domestic Screening and Treatment,” 3) “Overseas Albendazole and Ivermectin” presumptive treatment, and 4) “Overseas Albendazole and Domestic Screening for *Strongyloides*”. Markov transition state models were used to estimate long-term effects of parasitic infections. Health outcome measures (four parasites) included outpatient cases, hospitalizations, deaths, life years, and quality-adjusted life years (QALYs).

**Results:**

The “No Program” option is the least expensive ($165,923 per cohort) and least effective option (145 outpatient cases, 4.0 hospitalizations, and 0.67 deaths discounted over a 60-year period for a one-year cohort). The “Overseas Albendazole and Ivermectin” option ($418,824) is less expensive than “Domestic Screening and Treatment” ($3,832,572) or “Overseas Albendazole and Domestic Screening for *Strongyloides*” ($2,182,483). According to the model outcomes, the most effective treatment option is “Overseas Albendazole and Ivermectin,” which reduces outpatient cases, deaths and hospitalization by around 80% at an estimated net cost of $458,718 per death averted, or $2,219/$24,036 per QALY/life year gained relative to “No Program”.

**Discussion:**

Overseas presumptive treatment for U.S.-bound refugees is a cost-effective intervention that is less expensive and at least as effective as domestic screening and treatment programs. The addition of ivermectin to albendazole reduces the prevalence of chronic strongyloidiasis and the probability of rare, but potentially fatal, disseminated strongyloidiasis.

## Author Summary

Intestinal parasites including hookworm, *Strongyloides stercoralis*, *Ascaris lumbricoides* and *Trichuris trichuria* have been found to be prevalent in refugee populations. More than 50,000 refugees resettle in the United States annually. Since parasitic disease associated with these infections are rare in the United States, there may be delays in diagnosis or improper treatment of refugees, especially for strongyloidiasis. The Centers for Disease Control and Prevention began overseas presumptive treatment programs in some refugee populations in 1999. We examined the cost and economic impact of an overseas presumptive treatment program with albendazole and ivermectin for Asian refugees. We found that the program costs about $15.12 per refugee and that the burden of intestinal parasites would be reduced by about 80% considering cases, hospitalizations, life years, and QALYs. Overall, the cost per QALY gained was $2,219 (95% CI: 600–29,500). Compared to an alternative program in which refugees would be screened and treated for these infections after arrival in the United States, the overseas presumptive treatment program is less expensive and at least as effective.

## Introduction

More than 50,000 refugees resettle in the United States annually [1] and often arrive with much higher prevalence rates of parasitic infections than is seen in the general U.S. population. [2–5] Since parasitic diseases are rare in the United States, there have been delays in diagnosis or inappropriate treatment [6–10] due in part to inadequate screening strategies. [11] The combination of high prevalence and inadequate domestic management can lead to significant morbidity or even mortality. [12] To better remediate intestinal parasitoses in arriving refugees, the Centers for Disease Control and Prevention (CDC) began overseas presumptive treatment programs in some refugee populations in 1999. Presumptive treatment is administered shortly before departure to the United States to minimize the risk of re-infection.

The initial programs included a single dose of albendazole for intestinal nematodes to all refugees departing from Asia and Africa and sulfadoxine-pyrimethamine for anti-malarial treatment among refugees departing from sub-Saharan Africa. In 2005, the recommendations were expanded to include either ivermectin or a 7-day course of albendazole for *Strongyloides stercoralis* infections. However, because of funding and logistic issues for procuring and delivering ivermectin, it has only been used in a pilot program. The full CDC guidelines are available at: www.cdc.gov/immigrantrefugeehealth/guidelines/refugee-guidelines.html.

The prevalence of helminth infection was estimated to be 18.6% among Asian refugees according to stool ova and parasite testing prior to the implementation of CDC’s presumptive treatment program [5]. Serologic testing of Asian refugees in the United States have also identified high rates (>20%) of *Strongyloides* infections. [2, 3] Although *Ascaris*, *Trichuris*, and hookworm infections are likely to self-resolve within 1 to 6 years after arrival [13], *Strongyloides* infections are likely to persist for many years after arrival.

Previously published work suggested that presumptive treatment of immigrants from high-burden countries with albendazole and ivermectin is cost-effective. [14, 15] However, these previous economic analyses had limited data on burden of disease in these populations. Newly available data [2, 3, 5, 6, 16, 17] allow for more accurate estimates of the burden and consequences of these infections in refugee populations. Additionally, earlier studies of the cost-effectiveness are out-of-date because of the changing cost of interventions and testing (e.g., the price for albendazole in the United States has increased from $2.64 per 400 mg in 2000 [14] to almost $120 in 2013). [18] In addition to lower overseas drug costs (frequently <$1.00 per dose), the logistics of overseas presumptive treatment allow for simultaneous interventions in large groups, decreasing the labor resources needed and allowing for better monitoring. We also have detailed cost estimates for overseas programs from ongoing or pilot programs. These new data can be used to quantify the economic impact of CDC’s presumptive treatment recommendations for Asian refugees, both the proposed, but largely unimplemented, use of ivermectin and the already-implemented albendazole. We selected the Asian refugee population for this study to focus on albendazole and ivermectin. The African refugee population was excluded from the analysis because they also receive praziquantel for schistosomiasis and coartem for malaria. However, an analysis of presumptive treatment of African refugees with albendazole and ivermectin is included in the appendix (section 9).

Our analytic objective was to quantify and compare the benefits and costs of overseas presumptive treatment with domestic screening and treatment programs and no intervention for intestinal parasitoses. The conditions included in the analysis were infections with *Ascaris lumbricoides*, *Trichuris trichiura*, hookworm, and *Strongyloides stercoralis*. Per CDC guidelines, domestic screening (stool samples + serology for *Strongyloides)* is unnecessary for refugees that receive presumptive treatment overseas unless they present with clinical symptoms or persistent eosinophilia.

## Methods

An economic decision tree model was developed to assess the costs and health impacts of four interventions:

1. “No Program“: refugees do not receive presumptive treatment overseas or screening for parasitic diseases after domestic resettlement.
2. “Domestic Screening and Treatment”: refugees are tested for parasitic infection after domestic resettlement and treated if necessary.
3. “Overseas Albendazole and Ivermectin”: refugees are presumptively treated with albendazole and ivermectin before departing for the United States. In this model, we assumed that 5% would require some testing because they would present for domestic follow-up exams with clinical symptoms (e.g., gastrointestinal).
4. “Overseas Albendazole and Domestic Screening for *Strongyloides*”: is a hybrid program in which refugees receive overseas presumptive albendazole treatment and are screened for *Strongyloides* through serologic testing during the domestic resettlement examination.

Two other alternatives are included in the appendix (Section 8), which evaluate the replacement of overseas presumptive treatment regimens prior to departure with domestic presumptive treatment programs after arrival. Domestic albendazole and ivermectin presumptive treatment has similar health outcomes as “Overseas Albendazole and Ivermectin”, but costs significantly more. Domestic presumptive albendazole and screening for *Strongyloides* has similar health outcomes as “Overseas Albendazole and Domestic Screening for *Strongyloides*”, but again costs significantly more.

We used Markov processes to estimate the probability of outpatient and inpatient treatment and to estimate the number of years with a parasitic infection. To estimate the number of QALYs, we assumed that refugee QALY weights would be similar to that of the average U.S. population by age. [19] Then, we subtracted a small QALY decrement (0.001) for each year spent with a parasitic infection from age-specific QALY weights for U.S. adults. [19] There is considerable uncertainty in this decrement, and it is evaluated in a sensitivity analysis. We discounted costs and health outcomes at 3% annually over a period of 60 years after arrival. In the model, we assumed that refugees died of causes unrelated to parasitic infections at a rate equivalent to that for all Asian Americans [20] because refugee-specific mortality rates were unavailable. We assumed the same drug efficacy for overseas and domestic treatment, but considered an adjustment to reduce overseas efficacy in the sensitivity analyses.

The average age and average number of Asian refugees were estimated using U.S. entry data from 2002–11. [1] Domestic screening and outpatient treatment costs were estimated based on expected clinical procedures valued at Medicare [21, 22] and private insurance [23] reimbursement rates. Presumptive treatment costs were estimated by the International Organization for Migration in 2013; the primary contracted health service provider for U.S.-bound refugees. Key assumptions used to estimate epidemiologic and economic parameters are summarized in Table 1, with more detailed information of all input parameters in S1 Table.

**Table 1:** Summary of key epidemiologic, economic, and demographic parameter estimation.

*Study population*	• Average annual cohort of 27,700 Asian refugees based on Department of Homeland Security data for 2002–11, primarily from Southeast Asia and the Middle East. • Assume 100% of refugees can be covered by presumptive treatment programs in the future, (currently, an estimated 85% of refugees travel from countries where presumptive treatment programs currently exist, the remaining 15% come from countries without presumptive treatment) • 90% of refugees present for comprehensive exams after arrival in the United States
*Epidemiologic parameter estimation*	• Test sensitivities for infections vary from 78% for hookworm to 96% for *Trichuris* based on two stool ova and parasite tests and one *Strongyloides* serologic test. [24, 25] • The estimated specificities were 100%, except for *Strongyloides* serology (92%). [24] • Albendazole effectiveness was estimated based on a meta-analysis that reported efficacy varied between 28% against *Trichuris* to 88% against *Ascaris* infections. [26] • Ivermectin efficacy was estimated to be 90% for a 2-day treatment regimen [27–29]. • The prevalence of hookworm, *Trichuris*, and *Ascaris* infections (0.56% to 2.8%) was estimated from a multiyear study of newly-arrived refugees conducted in the State of Minnesota after adjustment for presumptive treatment and test sensitivity [5] using the following equation: *True prevalence* = ((*Reported prevalence* + *Specificity*—1) / (*Sensitivity* + *Specificity*– 1)) / (1 –effectiveness). • The prevalence of *Strongyoides* infections (20%) was estimated using the median rate from a number of serologic studies conducted among relocated Asian refugees. [2, 3, 16, 17, 30] • Duration of infection in the absence of treatment was: hookworm 6 years, *Trichuris* 2 years, *Ascaris* 1 year, *Strongyloides* indefinite [13] • The annual probabilities of outpatient and inpatient cases given infection were estimated from two previous studies that estimated the incidence of inpatient and outpatient strongyloidiasis among immigrant populations in New York state and Barcelona, Spain. [14, 31] • The risk of death from inpatient strongyloidiasis was estimated to be 16.7%. [15] • We assumed that side effects of presumptive treatment would be minor and not of economic significance. [32]
*Cost analysis*	• “Domestic Screening and Treatment” assumed two stool ova and parasite tests and one serologic test for *Strongyloides* infection comprising 10% of a comprehensive exam. Unit costs were estimated using two sets of reimbursement rates: 1) the Physician’s Fee and Coding Guide [23] and 2) the Medicare Physician Payment and Clinical Lab Fee Schedules [21, 22]. • Persons with positive test results required a follow-up visit and albendazole for hookworm, *Trichuris*, or *Ascaris* infections or ivermectin for *Strongyloides*. • Medicine costs were estimated from Red Book(R) data [18], assuming average dosages of 400 mg for albendazole and 18 mg for ivermectin. • Outpatient treatment costs were based on an assumed battery of tests and two outpatient visits. • Strongyloidiasis hospitalization costs were estimated from the 2006–2010 National Inpatient Sample data (ICD code 127.2) [33] and adjusted to 2013 USD using the Medical Consumer Price Index [34]. • Opportunity costs were estimated based on screening time (2 hrs.), treatment given positive test (1 hr.), outpatient treatment (1 day), and hospitalization (10 days). • The value of time was estimated using US GDP per capita-hr. estimates ($5.84 per hr.). [35] • Cost estimates for *Strongyloides*-only screening for the “Overseas Albendazole and Domestic Screening for *Strongyloides*” program omitted stool ova and parasite testing costs and assumed that 5% of the comprehensive exam time would be required for serologic testing. • Overseas presumptive treatment costs were estimated by IOM in 2013 and included medicine, delivery, administrative, and overhead cost data from three IOM sites.

The incremental societal costs per case, hospitalization, and death averted or per life year or quality adjusted life year (QALY) gained were: ***C****_program*_2_ + ***C****_illness*_2_ − [***C****_program*_1_ + ***C****_illness*_1_] divided by the difference in cases, life years, QALYs, hospitalizations, or deaths (e.g., [*Cases*_1_ − *Cases*_2_] or [*QALYs*_2_ − *QALYs*_1_) for these programs. The QALY calculation excluded illness opportunity costs (e.g., time lost from work or other activities due to illness) from the net cost calculation in the numerator.

Although it was assumed that 100% of Asian refugees would have access to presumptive treatment for this analysis, at present, the overseas presumptive treatment program is not available in all countries from which refugees travel. Those who do not receive overseas presumptive treatment (e.g., no presumptive treatment program in country) would receive the same battery of tests and treatment as described in “Domestic Screening and Treatment”. A range of 75%-100% of refugees traveling from countries with overseas presumptive treatment programs was considered in the sensitivity analysis with the remaining refugees that travel from countries without presumptive treatment still requiring domestic screening and treatment. This limitation is examined in a sensitivity analysis. In addition, one-way sensitivity analyses were conducted by varying individual parameters across their uncertainty ranges while holding all other parameters at base case values. Multivariate probabilistic sensitivity analysis was based on Monte Carlo Simulation using simultaneous random draws for each uncertain variable using probability distributions summarized in S2 Table.

We used Treeage Pro 2012 (Williamstown, MA) to build decision tree and Markov models and to conduct one-way and multivariate sensitivity analyses. Treeage output data were exported to Microsoft Excel (Redmond, WA) to create summary tables and figures. The Treeage model is available as a supplemental file.

This activity uses previously collected aggregate data and does not involve contact with human subjects. This analysis was ruled exempt from human subjects review by CDC.

## Results

### Health impact and costs

The “No Program” option is the least expensive, costing $165,369 per cohort of 27,700 refugees, $5.99 per refugee (Table 2). Program costs are zero and only outpatient (all four parasites) or inpatient strongyloidiasis cases incur costs. The “Overseas Albendazole and Ivermectin” option ($418,824 or $15.12 per refugee) is less expensive than “Domestic Screening and Treatment” ($3,832,572 or $138.36 per refugee). The “Overseas Albendazole and Domestic Screening for Strongyloides” option is intermediate in cost ($2,182,483 or $78.79 per refugee). The cost of “Overseas Albendazole and Ivermectin” includes overseas presumptive treatment program costs ($269,244) and illness costs ($31,855). In addition, “Overseas Albendazole and Ivermectin” includes domestic costs associated with the need to perform stool ova and parasite testing for 5% of refugees treated overseas with albendazole and ivermectin ($108,030).

**Table 2:** Estimated costs and health outcomes for parasite control programs among Asian refugees, 27,700 annual cohort.

	No program	Overseas Albendazole and Ivermectin	Domestic Screening and Treatment	Overseas Albendazole and Domestic Screening for *Strongyloides*
**Costs, 2013 USD**				
**Total costs (27,700 refugees)**	**$165,923**	**$418,824**	**$3,832,572**	**$2,182,483**
**Component Costs:**				
*Program (27*,*700 refugees)*	*$0*	*$386*,*969*	*$3*,*788*,*252*	*$2*,*138*,*163*
Health department for screening and treatment	$0	$108,030	$3,572,192	$1,988,029
Overseas presumptive treatment	$0	$269,244	$0	$79,776
Refugee opportunity (screening and treatment)	$0	$9,695	$216,060	$70,358
*Illness (27*,*700 refugees)*	*$165*,*923*	*$31*,*855*	*$44*,*320*	*$44*,*320*
Treatment (payments)	$139,885	$26,869	$37,395	$37,395
Treatment (opportunity)	$26,038	$4,986	$6,925	$6,925
**Cost per refugee, 2013 USD:**				
*Total* ^a^	*$5*.*99*	*$15*.*12*	*$138*.*36*	*$78*.*79*
Program ^b^	$0.00	$13.97	$136.76	$77.19
Illness ^c^^,^^d^	$5.99	$1.15	$1.60	$1.60
**Health outcomes (discounted):** ^**d**^				
Outpatient cases	145	29	40	39
Hospitalizations	4.0	0.8	1.1	1.1
Deaths	0.67	0.13	0.18	0.18
Life years	700,526	700,536	700,535	700,535
QALYs	605,253	605,377	605,366	605,366
**Economic outcomes relative to “No Program” expressed in Net Costs Per Category:**				
Case averted		2,146	34,907	18,483
Hospitalization averted		76,606	1,238,977	680,735
Death averted		458,718	7,419,026	4,076,259
Life year gained		24,036	388,754	213,594
QALY gained		2,219	32,706	18,167
QALY gained, 95% credibility interval ^e^		600–29,500	6,000–168,000	3,300–89,000

^a^ Total cost per refugee is calculated from the total (program + illness) cost divided by the cohort size (n = 27,700). This total may not be equal to the sum of program cost per refugee + illness cost per refugee because of rounding differences.

^b^ Program cost per refugee is calculated from the program cost divided by the cohort size (n = 27,700).

^c^ Illness cost per refugee is calculated from the illness cost divided by the cohort size (n = 27,700).

^d^ Cost and outcomes are discounted at 3% year over a 60-year period after arrival.

^e^ These credibility intervals are based on 10,000 random parameter draws using Monte Carlo Simulation (See S1 Appendix for details).

For “No Program”, our discounted estimates were 145 outpatient cases, 4.0 hospitalizations, and 0.67 deaths over a 60-year post-arrival period of a one-year cohort of 27,700 Asian refugees. The parasite that has the greatest morbidity and mortality potential is *Strongyloides*, which we estimate causes 96% of outpatient cases and all hospitalizations and deaths (see S1 Appendix for details).

Considering the impact on QALYs caused by all four parasites, the most effective treatment option is “Overseas Albendazole and Ivermectin”, which would reduce disease burden by an estimated 80%. The screening programs would be less effective (~72%), primarily because of the sensitivity of available diagnostics. For the base case analysis, “Overseas Albendazole and Ivermectin” is both less expensive and results in better health outcomes relative to either of the programs incorporating domestic screening and treatment.

Relative to “No Program”, “Overseas Albendazole and Ivermectin” would cost an estimated $2,146 per case, $76,606 per hospitalization and $458,718 per death averted. The cost per life year and QALY gained are $24,036 and $2,219 respectively. In comparison, the cost per QALY gained is $18,312 for “Overseas Albendazole and Domestic Screening for *Strongyloides*” and $32,706 for “Domestic Screening and Treatment” relative to “No Program”.

### Sensitivity analysis

**Fig 1:**
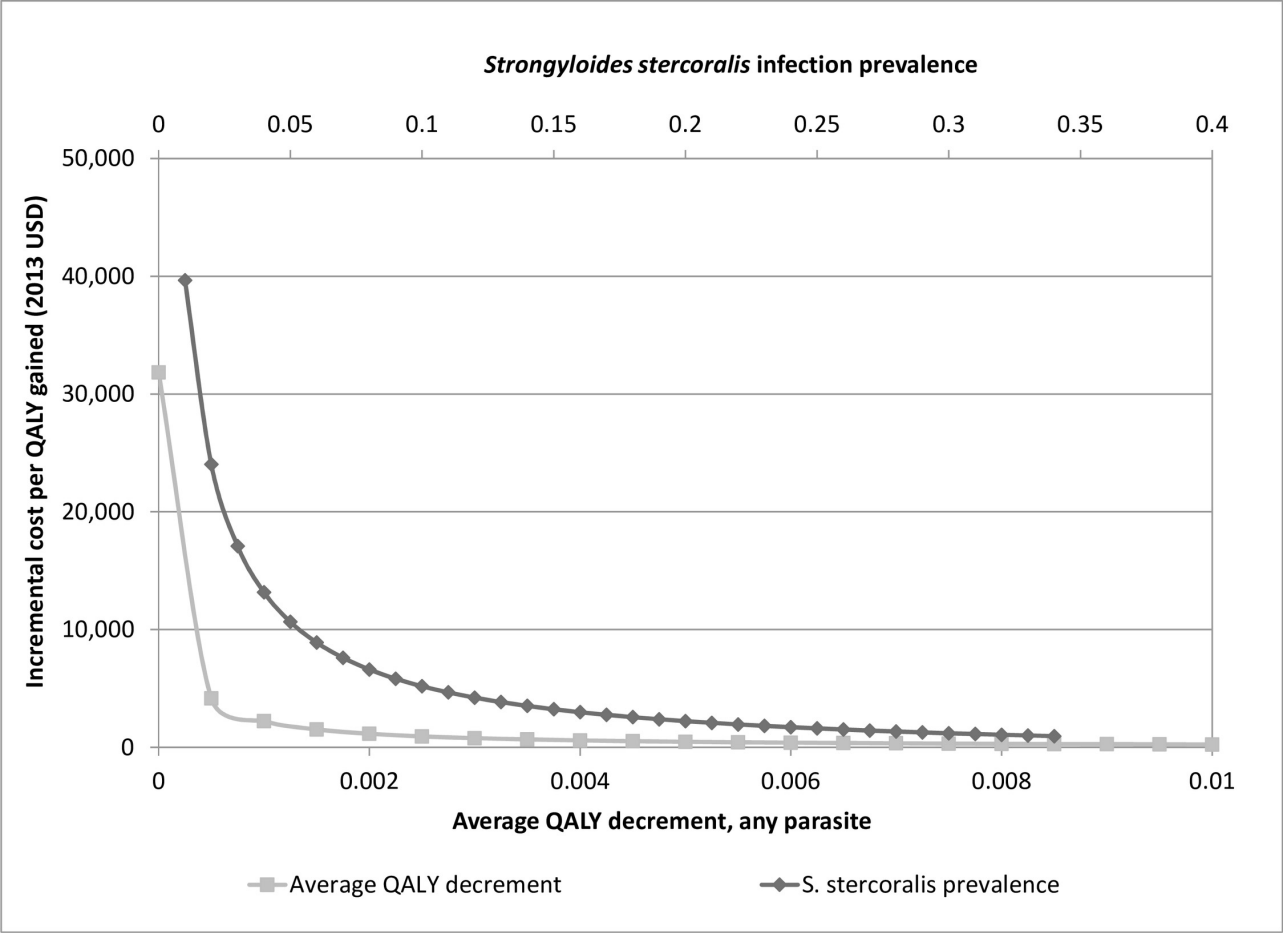
One-way sensitivity analyses of *Strongyloides* infection prevalence and QALY decrement on the cost per QALY gained, “Overseas Albendazole and Ivermectin” compared to “No Program” (2013 USD).

Fig 1 shows the expected cost per QALY gained as functions of *Strongyloides* prevalence and QALY decrement for infected refugees (holding all other parameters at base case values). At very low QALY decrements, the cost per QALY gained is similar to the cost per life year gained, ~$32,000, and decreases rapidly as the QALY decrement increases. For *Strongyloides* prevalence, the cost per QALY gained starts at about $40,000 at 1% prevalence and decreases to less than $18,000 at prevalence rates greater than 3%. A value of $50,000 per QALY gained, has commonly been cited as a reasonable threshold for identifying cost-effective interventions.[36] Considering the cost per QALY gained is $2,219 at the baseline prevalence estimate of 20% and remains below $50,000 at prevalence rates as low as 1%, this sensitivity analysis suggests that “Overseas Albendazole and Ivermectin” will probably be considered cost-effective.

**Fig 2:**
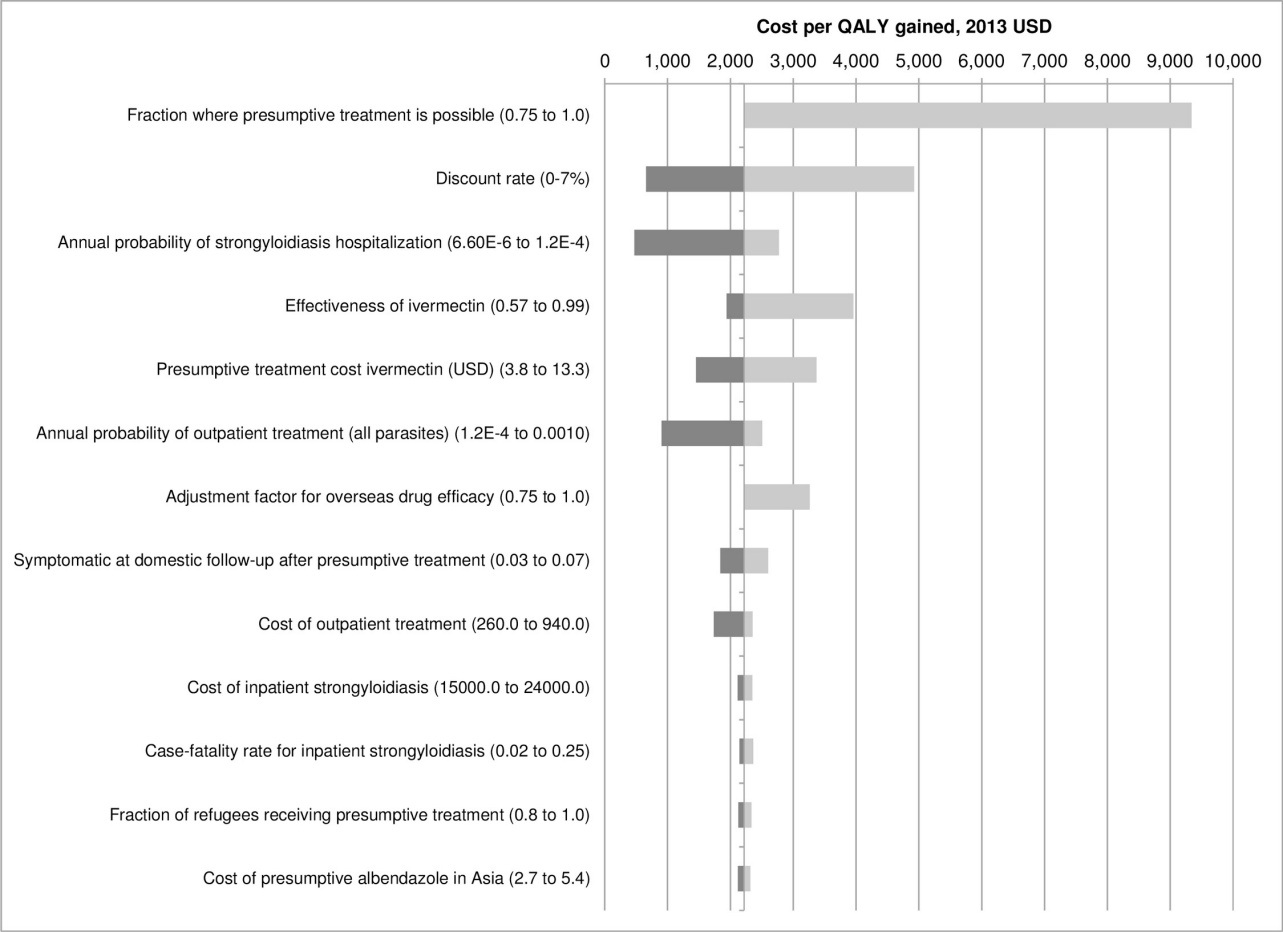
One-way sensitivity analysis of cost per QALY gained, 2013 USD ^a^. ^a^ The tornado diagram is a series one-way sensitivity analyses in which parameters are varied (one at a time across their uncertainty ranges while holding all other parameters at their base case value).

Fig 2 is a tornado diagram showing the minimum and maximum cost per QALY gained based on changing one parameter at a time according to the minimum and maximum values shown in S1 Table. After the QALY decrement and *Strongyloides* prevalence, the parameters with the most influence (in decreasing importance) are 1) the probability that refugees arrive from a country where IOM provides presumptive treatment, 2) discount rate, 3) annual probability of strongyloidiasis hospitalization, 4) ivermectin efficacy, 5) cost of ivermectin presumptive treatment overseas, 6) annual probability of an outpatient strongyloidiasis case, and 7) an adjustment factor for differences in relative overseas and domestic drug efficacy. The maximum cost per QALY gained remains less than $10,000 for all of these parameters. Of note, the cost per QALY gained varies from $472 using the upper bound of the range for the annual probability of hospitalization (0.00012) given *Strongyloides* infection to $2,775 at the lower bound (0.0000066). The case fatality rate among hospitalized cases is less important, varying from $2,146 at 2% mortality during hospitalization to $2,366 at 25%. In case overseas presumptive treatment is less effective than domestic treatment, a correction factor of 75% is also assessed in the one-way sensitivity analyses and results in a cost per QALY gained estimate of $3,265. The uncertainty in efficacy of ivermectin against *Strongyloides* (57–99%) results in a range of cost per QALY estimates from $1,943 to $3,959.

If the presumptive treatment program were not available to all refugees and some refugees had to go through domestic screening and treatment, the combined cost of the overseas presumptive treatment and domestic screening and treatment would increase compared to the ideal “Overseas Albendazole and Ivermectin” program in which presumptive treatment is possible for all refugees. Assuming that presumptive treatment could only be provided to 75% of refugees before they relocate to the United States and that the remaining 25% would go through domestic screening and treatment, the cost per QALY gained would increase from $2,219 to about $9,300.

**Fig 3:**
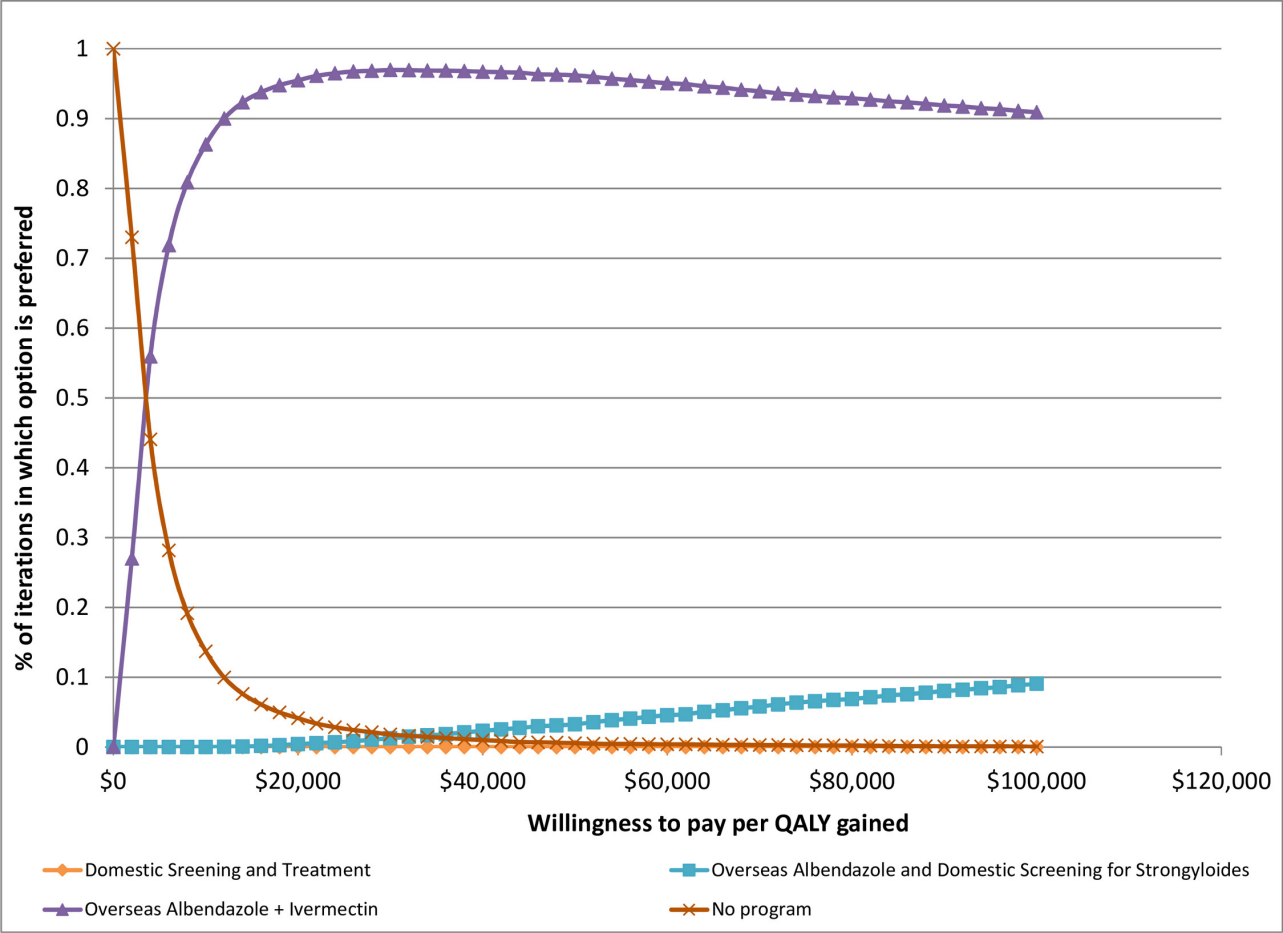
Cost-effectiveness acceptability curve, fraction of Monte Carlo Simulation iterations in which option is preferred as a function of willingness to pay per QALY gained.

Credibility intervals (95%) can be estimated from the results of the multivariate Monte Carlo Simulations. Relative to “No Program”, the cost per QALY gained varies from $600 to $29,500 for “Overseas Albendazole and Ivermectin” and from $6,000 to $168,000 for “Domestic Screening and Treatment.” A cost-effectiveness acceptability curve is shown in Fig 3. This figure shows the probability that each option is preferred as a function of decision makers’ willingness to pay per QALY gained. At low willingness to pay (<$3,000), the “No Program” option is preferred because this is always the lowest cost option. At willingness to pay per QALY gained > $12,000, the “Overseas Albendazole and Ivermectin” program is preferred in over 90% of the simulations.

## Discussion

Overseas presumptive treatment with albendazole and ivermectin is a cost-effective intervention for improving the health of refugees prior to arrival in the United States. Across a range of parameter estimates, the cost per QALY gained is less than $10,000 compared with waiting for symptomatic refugees to present with illness. Compared with domestic screening programs, overseas presumptive treatment is both less expensive and more effective.

Although all intestinal parasites can detrimentally affect health in refugees, *Strongyloides* infection presents the greatest threat to refugees due to 1) high infection prevalence in adults, 2) the potential for lifelong infection, and 3) serious sequelae including death, if infected refugees develop dissemination or hyperinfection syndrome due to an immunocompromised state. Assuming the same QALY decrement and probability of outpatient strongyloidiasis treatment as for other parasitic diseases, the potential health burden (cases, QALYs) is more than 20 times greater than for hookworm, *Trichuris*, and *Ascaris* infections combined. However, until recently, CDC’s presumptive treatment program has relied on a single dose of albendazole, which would have little effect on *Strongyloides* prevalence. Further, *Stongyloides* is not currently targeted as part of any soil transmitted helminth control programs in endemic countries.

This analysis has some limitations. It is difficult to quantify the QALY burden associated with chronic intestinal parasitism, especially in people outside an endemic area. Most people would not be aware that they were infected and would have vague symptoms from conditions associated with infection (e.g., anemia with hookworm, abdominal complaints with strongyloidiasis).[4] Although this study emphasizes *Stronglyloides*, *Trichuris*, *Ascaris* and hookworm infections, albendazole and ivermectin may impact other infections such as other helminths (e.g. enterobius) and ectoparasites (e.g. scabies). This would lead to underestimation of the health impacts and QALYs gained relative to “No Program”. However, as shown in Fig 1, even limiting the analysis to four helminths and using very conservative QALY decrements <0.0005 would make presumptive treatment cost-effective.

In addition, although there are CDC recommendations, we had no actual data regarding the types and frequencies of diagnostic tests given to refugees at domestic follow-up examination. In accordance with CDC guidelines we assumed that at least two stool ova and parasite examinations and one *Strongyloides* serologic test would be necessary, but examinations may include fewer, or more likely, more extensive diagnostic testing. Finally, we have very limited data on the probability that infected persons seek treatment either as outpatients or inpatients. Strongyloidiasis-related hospitalizations may be underreported because physicians unfamiliar with strongyloidiasis may report acute respiratory failure or gram-negative sepsis as hospitalization causes. [10] When data were unavailable, we tried to use conservative estimates. Nonetheless, these parameters had a small effect on the uncertainty in cost per QALY estimates as shown in Fig 2. Another limitation is that this analysis is limited to scenarios in which it is possible to schedule refugee departures from countries with high prevalence rates of helminth infections to the United States. Such programs cannot be implemented for persons already in the United States seeking asylum status. Similarly, such programs could not be implemented among the unprecedented numbers of refugees entering Europe during 2015–16. [37]

The analysis assumes that domestic physicians will use documented overseas presumptive treatment to forego refugee screening. This requires both adequate documentation of overseas treatment and physician knowledge and acceptance of treatment as sufficient (assuming refugees have no other symptoms consistent with intestinal parasitosis at the post-arrival comprehensive examination). While significant progress has been made, the transmission of overseas refugee medical treatment documentation to domestic physicians remains a work in progress especially as intervention coverage expands and programs evolve. Documentation issues should become easier over time.

In conclusion, the high cost of drugs and diagnostic tests in the United States, inconsistencies in the domestic approach to intestinal parasitosis, and limited provider knowledge about these neglected tropical diseases make overseas presumptive treatment of U.S.-bound refugees a good investment. The addition of ivermectin to albendazole presumptive treatment will improve the health of newly arriving refugees and reduce their long-term risk of complicated strongyloidiasis and hospitalization.

## Supporting Information

S1 Appendix Online Appendix. (DOCX)

S1 Table Epidemiological and economic input parameters. (DOCX)

S2 Table Estimated means and standard deviations for each uncertain parameter included in the analysis. (DOCX)

S1 File Online Treeage analysis file. (TREX)
