# Supplementary material for: Economic Analysis of the Impact of Overseas and Domestic Treatment and Screening Options for Intestinal Helminth Infection among US-Bound Refugees from Asia
Source: PLoS Negl Trop Dis. 2016 Aug 10;10(8):e0004910. doi: 10.1371/journal.pntd.0004910 (PMC4980012; doi:10.1371/journal.pntd.0004910)
Supplement: S1 Appendix — (DOCX) [file pntd.0004910.s001.docx]

**Appendix for Economic analysis of the impact of overseas and domestic treatment and screening options for intestinal helminth infection among US-bound refugees from Asia**

Brian Maskery,^1^ PhD, MS; Margaret S. Coleman,^1^ PhD; Michelle Weinberg, ^1^ MD; Weigong Zhou,^1^ MD, PhD; Lisa Rotz,^1^ MD; Alexander Klosovsky,^2^ MD; Paul T. Cantey,^3^ MD, MPH; LeAnne M. Fox, MD, MPH, DTM&H ^3^; Martin S. Cetron, MD^1^; and William Stauffer,^1,4^ MD, MSPH, DTM&H

^1^ Division of Global Migration and Quarantine, Centers for Disease Control and Prevention, Atlanta, GA, USA

^2^ International Organization for Migration

^3^ Division of Parasitic Diseases and Malaria, Centers for Disease Control and Prevention, Atlanta, GA, USA

^4^ Division of Infectious Diseases and International Medicine, University of Minnesota

Contents

[Overview 2](#_Toc444121381)

[1. Decision Tree and Markov models 13](#_Toc444121382)

[2. Epidemiological parameter estimates 17](#_Toc444121383)

[3. Comprehensive exam costs for intestinal parasite screening 22](#_Toc444121384)

[4. Outpatient and inpatient treatment cost estimates 28](#_Toc444121385)

[5. Overseas presumptive treatment cost estimates 32](#_Toc444121386)

[6. Probability distributions 33](#_Toc444121387)

[7. Baseline disease burden by parasite 36](#_Toc444121388)

[7. Additional sensitivity analysis 38](#_Toc444121389)

## Overview

This Appendix provides the complete details of the methods used in “Economic analysis of the impact of overseas and domestic treatment and screening options for intestinal helminth infection among US-bound refugees from Asia.” An economic decision tree model was developed to assess the costs and health impacts of four interventions for four parasites (*Ascaris lumbricoides*, *Trichuris trichiura*, hookworm, and *Strongyloides stercoralis*): 1) “No Program”, 2) “Domestic Screening and Treatment”, 3) “Overseas Albendazole and Ivermectin” presumptive treatment, and 4) “Overseas Albendazole and Domestic Screening for *Strongyloides*”. The distributions of age and country of origin of resettled refugees were averaged from multiple years of data [[1](#_ENREF_1)]. Markov transition state models were used to estimate the long term effects of *Strongyloides* infections that may remain asymptomatic for many years or may cause severe disease, known as hyperinfection [[2](#_ENREF_2)]. The costs of domestic screening and outpatient treatment were estimated based on expected clinical procedures valued at a weighted average of Medicare [[3](#_ENREF_3)] and private insurance reimbursement rates. Presumptive treatment costs were estimated by the International Organization for Migration (IOM); currently the primary health service provider for US-bound refugees. For each uncertain parameter estimate, we provided our best estimate based on what we believe to be the most likely value as well as minimum and maximum values (Table S1). These uncertainty ranges are used in univariate and multivariate sensitivity analyses.

*Study population*

From 2002-11, about 51,500 refugees relocated to the United States each year. The refugee population included an average of 27,700 from Asian countries, with most (94%) from: Myanmar (31.9%), Iraq (22.7%), Bhutan (16.7%), Iran (11.6%), Laos (5.7%), and Vietnam (5.6%). [[4](#_ENREF_4)] The median age of Asian refugees was 24 years. [[4](#_ENREF_4)]

The CDC works with IOM and other partners to coordinate health care and travel logistics for US-bound refugees. At present, IOM delivers presumptive albendazole treatment to US-bound refugees for which treatment is not contraindicated (i.e. pregnant women and children less than 1 year of age). In most years, IOM provides medical examinations and treatments to 80-90% of US-bound refugees. The percentage of US-bound refugees cared for by IOM varies from country to country and year to year. Refugees traveling from countries without an IOM presence do not currently receive presumptive treatment, but CDC’s goal is to provide presumptive treatment to all refugees. Thus, 100% is used as the baseline estimate. As part of the sensitivity analysis, the coverage rate was varied between 75% and 100%.

After arrival in the United States, all refugees are recommended to present for comprehensive medical evaluations. Data from the Office of Refugee Resettlement indicated that more than 90% of refugees from reporting states present for such exams; however, the tests included in the exams are not reported to CDC and vary depending on refugees’ final destinations (Curi Kim, CDC Division of Global Migration and Quarantine, liaison to Office of Refugee Resettlement, personal communication).

*Decision tree model*

Four program options were evaluated in the decision tree model: 1) “No Program”, 2) “Domestic Screening and Treatment”, 3) “Overseas Albendazole and Ivermectin’ presumptive treatment and 4) “Overseas Albendazole and Domestic Screening for *Strongyloides*”. For each branch/program option, refugees were subdivided by a) no infection or being infected with: b) hookworm, c) *Ascaris*, d) *Trichuris*, or e) *Strongyloides*. We did not account for simultaneous infections due to lack of data on the severity of multiple versus single infections.

The simplest branch is the baseline ‘No Program,’ where refugees receive no presumptive treatment overseas nor any domestic parasitic screening post resettlement. Thus, there are no program costs, and only domestic treatment costs if refugees present for treatment after arrival.

The “Domestic Screening and Treatment” branch is a program in which there is no presumptive treatment overseas. Instead, refugees receive a battery of tests during their domestic comprehensive medical exams. Some refugees (10%) do not present for comprehensive exams and are as likely to develop symptomatic illness as those in the “No Program” branch. Our analysis also includes consideration of both false positive (only for *Strongyloides*) and false negative (for all parasites) diagnostic test results. If refugees receive false positive results, they would incur the costs associated with medication despite not being infected. If they receive false negative results, they would not receive treatment for infections. Potential treatment failures are also included in the model.

The “Overseas Albendazole and Ivermectin’ presumptive treatment branch is a program where refugees are treated with both drugs immediately before their departure for the United States. Among IOM-treated refugees, some fraction may not receive presumptive treatment due to drug shortages or other logistical issues. We assumed that 90% of refugees traveling from countries with presumptive treatment programs would actually be treated and that the remaining 10% would be medically ineligible (e.g., pregnant women) or would be missed for other reasons. Drug efficacies were assumed to be the same overseas and in the United States. However, we added a correction factor in the sensitivity analysis to account for potential lower quality overseas drugs or reinfection before departing for the United States.

“Overseas Albendazole and Domestic Screening for *Strongyloides*”’ is a hybrid program where refugees receive overseas presumptive treatment with albendazole, but not ivermectin. In this case, refugees would be screened for *Strongyloides* and potentially treated at their post-arrival comprehensive medical exams. A single dose of albendazole is not considered an effective treatment for *Strongyloides* infections*.*

Markov processes were used to estimate the probabilities of refugees receiving outpatient or inpatient treatment in the United States. A constant annual probability of treatment was assumed over each parasite’s infection duration (Table S1). *Strongyloides* was assumed to remain present for the lifetime of infected persons because it is the only parasite that can reproduce in its human host. If inpatient or outpatient treatment was sought, it was assumed that all parasites would be eliminated. The Markov model included an annual probability of death from background (non-parasite) causes to account for persons who die of other causes before seeking treatment for infection. We applied the background mortality rates for all Asian Americans because Asian refugee-specific mortality data were not available [[5](#_ENREF_5)]. In the baseline Markov model, a small QALY decrement (0.001) was subtracted for each year spent with untreated parasitic infection. The previous cost-effectiveness model of ivermectin presumptive treatment only applied QALY decrements to cases in which outpatient or inpatient treatment occurred. [[6](#_ENREF_6)] However, very large decrements were estimated for both inpatient (0.201) and outpatient cases 0.081). Our assumed decrement was much smaller than the DALY weight (0.03) used for children with hookworm, *Trichuris*, or *Ascaris* infections used in the Global Burden of Disease study [[7](#_ENREF_7)]. While QALYs measure quality of life and DALYs measure the disability associated with health conditions, an estimated QALY decrement should be roughly comparable to a DALY weight estimated for the updated Global Burden of Disease study. The Global Burden of Disease study does not report a DALY weight for *Strongyloides* or an adult-specific *Ascaris, Trichuris*, or hookworm for adults. Although disability from growth impairment is likely less for adults than for children with these conditions, we used a much smaller QALY decrement for our baseline analysis.

*Epidemiological parameter estimation*

The epidemiological parameters are summarized in Table S1. Calculations and details are presented in Section 2 of this appendix.

Stool ova and parasite tests are used to diagnose hookworm, *Trichuris* and *Ascaris* infections. The sensitivity of stool ova and parasite tests for diagnosis of hookworm, *Trichuris* and *Ascaris* can be improved by collecting stool samples on multiple days since parasite shedding varies day-to-day. The sensitivity and specificity of screening tests were estimated assuming that two stool ova and parasite tests and one *Strongyloides* serologic test would be performed at all initial comprehensive medical exams in the absence of presumptive treatment. The estimated sensitivities of these tests varied by parasite from 78% to 96% (Table S1) [[8-13](#_ENREF_8)]. Sensitivities were estimated using on a meta-analysis tests for these three helminths based on the assumption that the formol-ether concentration would be used. [[13](#_ENREF_13), [14](#_ENREF_14)] The estimated specificities were assumed to be 100%, except for *Strongyloides* serology (92%) [[12](#_ENREF_12)].

Albendazole effectiveness has been evaluated in many randomized trials in multiple endemic countries. A meta-analysis estimated that efficacy varied between 28% against *Trichuris* to 88% against *Ascaris* [[15](#_ENREF_15)]. Since these studies occurred in endemic areas, treated individuals may have been re-exposed after treatment and before retesting. Thus, these estimates should be conservative for U.S.-bound refugees. An upper bound efficacy estimate was based on reported rates of hookworm, *Ascaris*, and *Trichuris* infections before and after the introduction of overseas presumptive treatment with albendazole. Ivermectin is currently the drug of choice for the treatment of intestinal strongyloidiasis in the absence of dissemination or immunosuppression [[16](#_ENREF_16)]. Efficacy rates have been observed between 57-100%. The lower bound was based on a study that used serologic testing to determine eradication of *Strongloides* [[17](#_ENREF_17)], but for which only a single dose of ivermectin was administered. At least one study has shown that a two dose regimen is more effective. [[18](#_ENREF_18)] The lowest reported efficacy for the CDC-recommended 2-day treatment regimen is 93%. [[17-19](#_ENREF_17)]

The prevalence of hookworm, *Trichuris*, and *Ascaris* infections was estimated from a multiyear study of newly-arrived refugees conducted in the state of Minnesota [[20](#_ENREF_20)]. Since this study includes data from refugees that may have been treated presumptively with albendazole, it was necessary to adjust for drug effectiveness, sensitivity and specificity using the following equation: *True_prevalence* = ((*Reported_prevalence* + *Specificity* - 1) / (*Sensitivity* + *Specificity* – 1)) / (1 – effectiveness).

The prevalence of *Strongyloides* infections was estimated using the median rate from a number of studies using serologic testing and conducted among relocated Asian refugees [[21-25](#_ENREF_21)]. The median estimate was adjusted for sensitivity and specificity, but not drug efficacy because ivermectin presumptive treatment had not been implemented when the studies were undertaken.

The annual probabilities of outpatient and inpatient cases given infection were estimated from two previous studies that estimated the incidence of inpatient and outpatient strongyloidiasis among immigrant populations in New York state and Barcelona, Spain. [[6](#_ENREF_6), [26](#_ENREF_26)] Both studies found that strongyloidiasis cases were most commonly diagnosed as a result of unexplained eosinophilia (64% in the Barcelona study).

The annual probability of outpatient and inpatient cases among refugees with *Ascaris*, *Trichuris*, and hookworm infections was assumed to be the same as for *Strongyloides* infections because most of these patients also present with unexplained eosinophilia and because we had no data. However, these parasites cannot replicate within the human host and the duration of infection is limited to 1 year for *Ascaris*, 1-2 years for *Trichuris* and 5-7 years for hookworm (Table S1).

The risk of death from *Ascaris*, hookworm and/or *Trichuris* infections is assumed to be zero on the assumption that refugees will not be further exposed to these parasites in the United States. Therefore, parasite intensity would decrease over time even without treatment. The risk of death from inpatient strongyloidiasis was estimated to be 16.7% [[27](#_ENREF_27)] because, by the time most patients were admitted to hospitals and diagnosed with strongyloidiasis, they were extremely ill. [[28](#_ENREF_28)]

We assumed that side effects of presumptive treatment would be minor and not of economic significance because there is little or no evidence that side effects require treatment. A recent study showed that none of 19,000 triple drug recipients (albendazole, ivermectin and praziquantel) in Zanzibar required a visit to a health center due to side effects. [[29](#_ENREF_29)]

*Cost analysis*

The economic cost estimation is described in more detail in Sections 3-5 of this appendix. For “Domestic Screening and Treatment”, we assumed refugees would undergo two stool ova and parasite tests and one serologic test for *Strongyloides* infection. This testing would be initiated within the context of a comprehensive medical examination where intestinal parasite tests are just one set of tests. We assumed the intestinal parasite investigation would comprise 10% of the cost of the comprehensive exam and would require 0.25 hours of language translation services.

Unit costs were estimated using two sets of reimbursement rates: 1) the Physician’s Fee and Coding Guide and 2) the Medicare Physician Payment and Clinical Lab Fee Schedules. [[3](#_ENREF_3), [30](#_ENREF_30), [31](#_ENREF_31)] Language translation costs were estimated from the Bureau of Labor Statistics’ mean hourly wage for translators, $24.33 (Occupation Code: 27-3091). [[32](#_ENREF_32)] An additional assumed 33% was added to the hourly wage rate to cover employee benefits.

Persons with positive test results were assumed to return for a follow-up visit and to receive a prescription for medicine (albendazole for hookworm, *Trichuris*, or *Ascaris* or ivermectin for *Strongyloides* infections). Medicine costs were estimated using the Red Book(R) database [[33](#_ENREF_33)], assuming average dosages of 400mg for albendazole over one day and 18 mg over two days for ivermectin.

Outpatient treatment cost data were not available directly. Given the non-specific symptoms of parasitic infections, some patients may seek treatment multiple times before a diagnosis is made. We assumed that a tropical disease specialist may order a battery of tests and require two outpatient consultations to diagnose a patient. Strongyloidiasis hospitalization costs were estimated from the 2006-2011 National Inpatient Sample data (ICD code 127.2 as the primary diagnosis) [[34](#_ENREF_34)] and adjusted to 2013 USD using the Medical Consumer Price Index .

Opportunity costs were estimated based on the amount of time estimated to be required for screening (2 hrs. including two return trips with stool samples), treatment given positive test (1 hr.), outpatient treatment (1 day), and hospitalization (10 days). The value of time was estimated using US GDP per capita-hr. estimates ($5.84 per hr.) [[35](#_ENREF_35)]. For the sensitivity analysis, time was valued at the average hourly wage rate from the Bureau of Labor Statistics to estimate an upper bound (see Section 4 for details). The amount of time required for outpatient treatment and hospitalization are likely to be conservative, especially the opportunity cost of hospitalization since time required for rehabilitative therapy after hospitalization is not included.

Estimates of the cost of *Strongyloides*-only screening for the “Overseas Albendazole and Domestic Screening for *Strongyloides*” program omitted the costs of stool ova and parasites diagnostics and assumed that only 5% of the comprehensive exam (rather than 10%) would be devoted to intestinal parasite screening.

For ‘Overseas Ivermectin and Albendazole” or “Overseas Albendazole and Domestic Screening for *Strongyloides*” presumptive treatment costs were estimated by IOM and included medicine, delivery, administrative, and overhead costs based on data from three IOM sites in Thailand, Nepal, and Malaysia (see Section 5 for more details). We also assumed that some refugees would undergo stool ova and parasites testing despite overseas presumptive treatment because they may be infected with intestinal pathogens that are not susceptible to albendazole or ivermectin, or they may present with symptoms consistent with parasitic diseases that are also consistent with other types of diseases (e.g., diarrhea).

Table S1. Epidemiological and economic input parameters

| Description | Base case analysis | Low | High | Distri-bution ^a^ | Ref |
| --- | --- | --- | --- | --- | --- |
| ***Epidemiological parameters*** | | | | | |
| Baseline infection prevalence (without treatment) ^b^ | | | | | |
| Hookworm | 0.028 | 0.028 | 0.11 | B | [[20](#_ENREF_20)] |
| *Ascaris* | 0.0084 | 0.0084 | 0.017 | B | [[20](#_ENREF_20)] |
| *Trichuris* | 0.0056 | 0.0056 | 0.015 | B | [[20](#_ENREF_20)] |
| *Strongyloides* | 0.20 | 0.06 | 0.34 | B | [[21-25](#_ENREF_21)] |
| *Drug efficacy* ^b^ |  |  |  |  |  |
| Albendazole against hookworm | 0.72 | 0.72 | 0.93 | B | [[8](#_ENREF_8), [15](#_ENREF_15), [20](#_ENREF_20)] |
| Albendazole against *Trichuris* | 0.28 | 0.28 | 0.73 | B | [[8](#_ENREF_8), [15](#_ENREF_15), [20](#_ENREF_20)] |
| Albendazole against *Ascaris* | 0.88 | 0.88 | 0.94 | B | [[8](#_ENREF_8), [15](#_ENREF_15), [20](#_ENREF_20)] |
| Ivermectin against *Strongyloides* | 0.90 | 0.57 | 0.99 | B | [[17-19](#_ENREF_17)] |
| *Test sensitivity* ^b^ | | | | | |
| *Strongyloides* serologic test | 0.91 | 0.89 | 0.92 | B | [[12](#_ENREF_12)] |
| Two stool O&P for hookworm | 0.78 | 0.53 | 0.88 | B | [[13](#_ENREF_13), [14](#_ENREF_14)] |
| Two stool O&P for *Ascaris* | 0.81 | 0.57 | 0.95 | B | [[13](#_ENREF_13), [14](#_ENREF_14)] |
| Two stool O&P for *Trichuris* | 0.96 | 0.81 | 0.99 | B | [[13](#_ENREF_13), [14](#_ENREF_14)] |
| *Test specificity* ^b^ | | | | | |
| *Strongyloides* serologic test | 0.92 | 0.89 | 0.97 | B | [[9](#_ENREF_9), [11](#_ENREF_11), [12](#_ENREF_12)] |
| Two stool O&P for hookworm, *Ascaris*, or *Trichuris* | 1 | 1 | 1 | NA | Assumption |
| *Duration of infection* ^b^ | | | | | |
| Hookworm | 6 | 5 | 7 | G | [[36](#_ENREF_36)] |
| *Trichuris* | 2 | 1 | 2 | G | [[36](#_ENREF_36)] |
| *Ascaris* | 1 | 1 | 1 | N/A | [[36](#_ENREF_36)] |
| *Annual probability of seeking treatment given infection* ^b^ | | | | | |
| Outpatient visit for hookworm/ trichuriasis/ascariasis | 0.001 | 0.00012 | 0.005 | B | [[6](#_ENREF_6), [26](#_ENREF_26)] |
| Outpatient visit for strongyloidiasis | 0.001 | 0.00012 | 0.005 | B | [[6](#_ENREF_6), [26](#_ENREF_26)] |
| Inpatient strongyloidiasis | 2.9E-05 | 6.6E-06 | 1.2E-04 | B | [[6](#_ENREF_6), [26](#_ENREF_26)] |
| Case fatality rate for inpatient strongyloidiasis | 0.167 | 0.02 | 0.25 | B | [[27](#_ENREF_27)] |
| *Program parameters* | | | | | |
| Proportion of refugees receiving domestic comprehensive medical exam | 0.9 | 0.8 | 1.0 | U | Assumption |
| Probability refugees arrive from countries with presumptive treatment program | 1.00 | 0.75 | 1.00 | U | IOM report |
| Probability refugees receive presumptive treatment | 0.90 | 0.80 | 0.98 | U | Assumption |
| Probability stool O&P will be ordered given presumptive treatment | 0.05 | 0.03 | 0.07 | U | Assumption |
| Adjustment factor for overseas versus domestic treatment 0-1 | 1 | 0.75 | 1 | U | Assumption |
| *Demographics* | | | | | |
| Probability of death background rate | Varies with age | | | N/A | [[5](#_ENREF_5)] |
| Median age of refugees at arrival | 24 |  |  |  | [[4](#_ENREF_4)] |
| **Economic parameters** | | | | | |
| *U.S. cost estimates (2013 USD)* | | | | | |
| Screen for all nematodes ^c^ | 116 | 78 | 260 | G | [[3](#_ENREF_3), [30](#_ENREF_30), [31](#_ENREF_31)] |
| Screen for strongyloides, assuming albendazole presumptive treatment ^c^ | 54 | 38 | 113 | G | [[3](#_ENREF_3), [30](#_ENREF_30), [31](#_ENREF_31)] |
| Screen, assuming albendazole + ivermectin presumptive treatment ^c^ | 78 | 57 | 171 | G | [[3](#_ENREF_3), [30](#_ENREF_30), [31](#_ENREF_31)] |
| Albendazole treatment 400mg ^c^ | 173 | 152 | 234 | G | [[3](#_ENREF_3), [30](#_ENREF_30), [31](#_ENREF_31), [33](#_ENREF_33)] |
| Outpatient treatment for intestinal parasites (screening + albendazole cost) ^d^ | 494 | 345 | 1030 | G | [[3](#_ENREF_3), [30](#_ENREF_30), [31](#_ENREF_31), [33](#_ENREF_33)] |
| Outpatient treatment for intestinal parasites (screening + ivermectin cost) ^d^ | 408 | 259 | 940 | G | [[3](#_ENREF_3), [30](#_ENREF_30), [31](#_ENREF_31), [33](#_ENREF_33)] |
| Inpatient treatment for strongyloidiasis ^d^ | 20,000 | 15,000 | 24,000 | G | [[34](#_ENREF_34)] |
| Ivermectin treatment 18mg ^d^ | 87 | 66 | 150 | G | [[3](#_ENREF_3), [30](#_ENREF_30), [31](#_ENREF_31), [33](#_ENREF_33)] |
| *Overseas cost estimates (2013 USD) ^e^* | | | | | |
| Presumptive albendazole treatment in Asia | 3.2 | 2.7 | 3.7 | G | IOM data |
| Presumptive ivermectin treatment in Asia | 7.6 | 3.8 | 13.3 | G | IOM data |
| *Opportunity cost estimates (2013 USD)* | | | | | |
| Screening (all parasites) ^c^ | 7 | 7 | 22 | G | [[32](#_ENREF_32), [35](#_ENREF_35)] |
| Screening (*Strongyloides* only) ^c^ | 1 | 1 | 2.20 | G | [[32](#_ENREF_32), [35](#_ENREF_35)] |
| Treatment after screening ^c^ | 6 | 6 | 22 | G | [[32](#_ENREF_32), [35](#_ENREF_35)] |
| Outpatient cases ^d^ | 140 | 140 | 176 | G | [[32](#_ENREF_32), [35](#_ENREF_35)] |
| Inpatient strongyloidiasis ^d^ | 1,400 | 1,400 | 1,760 | G | [[32](#_ENREF_32), [34](#_ENREF_34), [35](#_ENREF_35)] |
| *QALY estimates* | | | | | |
| QALY decrement for *Strongyloides* infections | 0.001 | 0 | 0.01 | B | Assumption |
| QALY decrement for hookworm, *Ascaris*, *Trichuris* infections | 0.001 | 0 | 0.01 | B | Assumption |
| ^a^ Distribution types: U- uniform, B- beta, G- gamma (See Section 5 of appendix for more details)  ^b^ Details in Section 2 of Appendix  ^c^ Details in Section 3 of Appendix  ^d^ Details in Section 4 of Appendix  ^e^ Details in Section 5 of Appendix | | | | | |

## 1. Decision Tree and Markov models

*Decision tree models*

The costs and health impacts of alternative programs were estimated using a decision tree model that terminated with Markov processes. A simplified schematic of the decision tree model is shown in Figure S1, the complete decision tree model is available from the corresponding author. For each of the options, refugees begin in either an infected (with one of the four parasites) or uninfected state. For ‘Domestic Screening and Treatment’, refugees may or may not present for comprehensive examinations after arrival. If they do not follow up, they do not incur screening or treatment costs. However, infected refugees would remain infected. If they do follow-up, they are tested and the results may be:

1. True Positive (refugees are infected and treated)
2. False Positive (refugees are not infected, but are still treated)
3. True Negative (refuges are not infected and not treated)
4. False Negative (patients are infected, but not treated)

When patients are true positive or false positive, they will incur the cost of medications. If they are infected (true positive), the infection will be eliminated at the pathogen-specific efficacy listed in Table S1. If they are not infected (false positive), the refugees would be unnecessarily treated; however, this is unlikely to impose any costs beyond those associated with purchasing the medication. If refugees are infected, but test false negative, they will not receive medication and will remain infected.

For “Overseas Albendazole and Ivermectin”, we assumed that the program would be implemented for 100% of the refugee population. In the sensitivity analysis, we allowed for the possibility that it would not be possible to implement presumptive treatment programs in all Asian countries from which refugees travel and that some fraction of refugees would need to go through the screening and treatment protocol after arriving in the United States. Refugees were again subdivided by infection status. We assumed that 90% of refugees from countries with presumptive treatment programs would receive treatment overseas and that these medications would have the same efficacy as if the drugs were administered in the United States. Refugees end in one of five conditions after completing each decision process: 1) uninfected, 2) infected with hookworm, 3) infected with *Trichuris*, 4) infected with *Ascaris*, or 5) infected with *Strongyloides*.

For “No Program”, costs are only incurred among refugees that need to seek outpatient or inpatient treatment.

*Markov model*

For uninfected persons the Markov process is simple. Each year, they may die based on background mortality rates for Asian Americans reported in the CDC Wonder database [[5](#_ENREF_5)]. Otherwise, they remain in an uninfected stated with QALY weight based on the average estimated for the U.S. population. [[37](#_ENREF_37)]

For persons infected with hookworm, Trichuris, or Ascaris, Figure S2 shows that refugees start in the infected state. Each year they may: 1) die at the background rate; 2) seek outpatient treatment; 3) remain infected; or, 4) become clear of infection without treatment. There are no hospitalizations or deaths caused by these three parasites. After clearing the infection, there is zero probability of reinfection. For each year spent in the infected state, there is a small QALY decrement. In the baseline analysis, this decrement is 0.001 so the QALY weight for each year of infection is the baseline weight – 0.001. We assumed that the infection would clear without treatment after 1 year for *Ascaris*, 2 years for *Trichuris*, and 6 years for hookworm (Table S1) for the baseline scenario.

A schematic of the Markov model for persons infected with *Strongyloides* is shown in Figure S3. Each refugee again begins in an infected state. Each year, they may 1) remain infected, 2) receive outpatient treatment, 3) receive inpatient treatment, 4) they may die from other causes at the background mortality rate for all Asian Americans. If they are treated as inpatients there is a risk of death (Table S1). Anyone that received outpatient or inpatient treatment was assumed to clear the infection unless they died (i.e. there were no treatment failures).

Figure S1. Simplified schematic of decision tree model

^a^ Persons infected with one of *Ascaris*, *Trichuris*, *Strongyloides*, or hookworm

Figure S2. Schematic of Markov model for hookworm, *Trichuris*, and *Ascaris* infections

Death (unrelated to parasitic infection)

Infected (asymptomatic or subclinical

Infection cleared

Outpatient case

Figure S3. Schematic of Markov model for *Strongyloides* infections

Death (unrelated to strongyloidiasis)

Infected (asymptomatic or subclinical)

Infection cleared

Inpatient case

Outpatient case

Death (strongyloidiasis)

## 2. Epidemiological parameter estimates

**Hookworm, *Trichuris*, and *Ascaris***

Stool ova and parasite tests are used to diagnose hookworm, *Trichuris* and *Ascaris* infections. The sensitivity of these tests can be improved by collecting stool samples on multiple days since parasite shedding varies day-to-day. The sensitivity and specificity of screening tests in the United States were estimated by assuming that two stool ova and parasite tests would be performed at all initial comprehensive medical exams where Asian refugees did not receive presumptive treatment. The estimated sensitivities for each different parasite varied from 78% to 96% (Table S2) [[13](#_ENREF_13), [14](#_ENREF_14)]. Sensitivities were estimated using a meta-analysis of testing for these three helminths based on the assumption that formol-ether concentration would be used. [[13](#_ENREF_13), [14](#_ENREF_14)] Specificities were assumed to be 100%.

Albendazole effectiveness has been evaluated in many randomized trials in multiple endemic countries. A meta-analysis produced efficacy estimates of 28% against *Trichuris*, 72% against hookworm, and 88% against *Ascaris* [[15](#_ENREF_15)]. Since these studies occurred in endemic areas, treated individuals would likely have been re-exposed after treatment and before retesting. A comparison of prevalence before and after the introduction of presumptive albendazole treatment in US-bound refugees demonstrated a much greater effect; prevalence ratios (treated versus untreated) varied from 0.06 for *Ascaris*, 0.07 for hookworm and 0.27 for *Trichuris* [[20](#_ENREF_20)]. However, since these estimates were not obtained from a randomized control trial, it is not possible to distinguish declines in refugee baseline prevalence over time (even without treatment) compared to presumptive treatment-induced reductions in prevalence. Further, the prevalence of parasitic disease among refugees may have declined over time due to albendazole campaigns undertaken among non-refugee children and/or economic development in the host countries where refugees live. We used the meta-analysis efficacy estimates for the base case analysis, which we believe is very conservative since refugees will probably not be re-exposed to parasites after they arrive in the United States in contrast with the populations evaluated in the meta-analysis where the parasites are endemic. We used the pre- and post-introduction of presumptive treatment effectiveness estimates as an upper bound.

The prevalence rates of hookworm, *Trichuris*, and *Ascaris* were estimated from a multiyear study of newly-arrived refugees conducted in Minnesota [[20](#_ENREF_20)]. Infection prevalence can be estimated from the time period before albendazole presumptive treatment was initiated, 1999, in which case the estimate would be dated. Instead, we relied on more recent prevalence data, post-1999. To use this more recent data, we had to adjust for the effectiveness of overseas presumptive treatment. The reported prevalence rates must also be adjusted for the sensitivity and specificity of diagnostic tests, again assuming that two stool ova and parasite tests were performed. Thus, the expected prevalence before presumptive treatment may be calculated as True_prevalence = ((Reported_prevalence + Specificity - 1) / (Sensitivity + Specificity – 1)) / (1 – effectiveness).

Table S2. Hookworm, *Ascaris*, and *Trichuris* prevalence estimation after adjustment for drug efficacy and test sensitivity

|  | *Ascaris* | Hookworm | *Trichuris* | Source |
| --- | --- | --- | --- | --- |
| Prevalence after treatment | 0.001 | 0.007 | 0.4 | [[20](#_ENREF_20)] |
|  | | | | |
| Meta analysis efficacy | 0.88 | 0.72 | 0.28 | [[15](#_ENREF_15)] |
| Adjusted pre-treatment prevalence based on meta-analysis efficacy | 0.83 | 2.50 | 0.56 |  |
| Sensitivity adjustment (Best and Minimum prevalence estimate)^a^ | 0.84 | 2.84 | 0.56 | [[13](#_ENREF_13), [14](#_ENREF_14)] |
|  | | | | |
| Effectiveness based on pre and post treatment | 0.94 | 0.93 | 0.73 | [[20](#_ENREF_20)] |
| Adjusted pre-treatment prevalence based on effectiveness study | 1.67 | 10.00 | 1.48 |  |
| Sensitivity adjustment (Maximum prevalence estimate) ^a^ | 1.68 | 11.36 | 1.48 | [[10](#_ENREF_10)] |
| ^a^ Estimated sensitivity 0.81 for *Ascaris*, 0.99 for *Trichuris* and 0.78 for hookworm based on two stool O & P tests. | | | | |

Ivermectin 200µg/kg is currently the drug regimen of choice for the treatment of intestinal strongyloidiasis in the absence of dissemination or immunosuppression [[16](#_ENREF_16)]. A study of the treatment of chronic strongyloidiasis demonstrated a 100% (n=35) cure rate for the two-day ivermectin regimen verses a 77% (n=22) cure rate with the single ivermectin dose, and a 78% (n=25) cure rate with thiabendazole [[18](#_ENREF_18)]. Effectiveness of the two-dose regimen was slightly lower (93%) in a separate trial conducted in a population with co-morbidities [[19](#_ENREF_19)]. A lower efficacy rate (57-68%) was estimated when using serologic confirmation of *Strongyloides* elimination. However, only single-dose efficacy data was evaluated in this trial [[17](#_ENREF_17)]. Another study showed a lower cure rate with single-dose ivermectin (17/22) than with 2-dose ivermectin (35/35). [[18](#_ENREF_18)]

The prevalence of *Strongyloides* was estimated using the median rate from a number of serologic studies of Asian refugees resettled to the United States, Canada, and Australia [[21-25](#_ENREF_21)]. This median estimate was adjusted for sensitivity and specificity, but not drug efficacy because ivermectin presumptive treatment had not been implemented when the studies were undertaken. The raw data from studies using *Strongyloides* serology is summarized in Table S3. The fraction testing positive varied from 13% - 65%. However, these results did not account for the sensitivity and specificity of *Strongyloides* serologic tests. Study-specific sensitivity and specificity were likely to vary with the origins of the refugee population studied and the type of serologic test used. The most recent and most comprehensive study for *Strongyloides* serology sensitivity and specificity showed that commercially available tests had sensitivities of 89-92% and specificities of 89-97% [[12](#_ENREF_12)]. After adjustment using estimates of 91% sensitivity and 92% specificity, the prevalence estimates varied from 6%-69%. A Montreal study of immigrants from 1982-83 may be considered an outlier since estimates were almost twice as high as the next highest prevalence study, and the population may not be comparable to more recent refugee populations for a number of reasons. The median prevalence estimate after adjustment is 19.5% (assuming studies with separate estimates for Middle East vs. South and Southeast Asian refugees can be considered separate results). The sample size-weighted average is 19.6%. If the Montreal study was included, the median would increase to 23% and the sample-size-weighted average would increase to 26%. Among the five studies, only two were conducted in the United States; however, we believe that prevalence rates of refugees resettled to Canada and Australia would be similar to rates among refugees resettled in the United States.

Table S3. Prevalence of *Strongyloides stercoralis* infection in refugees in published studies using serologic detection methods

| Site | Sample size | South and Southeast Asia | | Sample size | Middle East | | Ref |
| --- | --- | --- | --- | --- | --- | --- | --- |
|  |  | Unadjusted | Adjusted ^a^ |  | Unadjusted | Adjusted ^a^ |  |
| Santa Clara County, CA 2001-10 | 67 | 27% | 23% | 136 | 21% | 16% | [[22](#_ENREF_22)] |
| Melbourne, Australia 2006-09 | 973 | 21% | 16% | NA | NA | NA | [[25](#_ENREF_25)] |
| Montreal, Canada 1982-83 | 232 | 65% | 69% | NA | NA | NA | [[24](#_ENREF_24)] |
| Syracuse, NY 2008-09 | 96 | 31% | 28% | 23 | 13% | 6% | [[23](#_ENREF_23)] |
| Melbourne, Australia 2000-02 | 230 | 36% | 34% | NA | NA | NA | [[21](#_ENREF_21)] |

^a^ The adjustments use an estimated sensitivity of 91% and a specificity of 92% in this formula ((Reported_prevalence + Specificity - 1) / (Sensitivity + Specificity – 1)).

Estimates of the incidence rates of outpatient and inpatient strongyloidiasis were available from two sources. Muennig et al. [[6](#_ENREF_6)] estimated an annual incidence rate of 42.5 outpatient visits per 100,000 persons infected with strongyloidiasis based on Medicaid data from the 1990s and an assumed *Strongyloides* infection prevalence of 10% among the U.S. immigrant population in New York City. Valerio et al. [[26](#_ENREF_26)] estimated an annual incidence of 10 cases per 100,000 immigrants in Barcelona, Spain. This estimate was not adjusted based on estimates of the prevalence of *Strongyloides* infection of the originating countries of the immigrant. If we assume an infection prevalence of 10%, the resulting incidence would be 100 outpatient cases per 100,000 immigrants with *Strongyloides* infection. Both studies reported that the majority of immigrants in each city were from Latin America, where previous studies included in a systematic review have shown that *Strongyloides* prevalence can vary between 1− 75% [[38](#_ENREF_38)]. Such a large range includes differences in both endemicity and research methods. Table S4 shows estimates of the annual probability of inpatient and outpatient treatment given a range of prevalence rates between 1% − 50% with a best estimate of 10% based on the assumptions in the Muennig study [[6](#_ENREF_6)]. The annual probability of being treated for strongyloidiasis as an outpatient was estimated to be between 8.5 and 500 cases per 100,000 infected person-years with a best estimate of 100 per 100,000. The annual probability of hospitalization was much lower and was estimated between 0.66 – 14 hospitalizations per 100,000 infected person-years with a best estimate of 2.9 per 100,000.

Table S4. Observed annual incidence of strongyloidiasis and estimated annual probability of treatment given variable infection prevalence rates

|  | Observed incidence | Annual probability of treatment given infection prevalence | | |
| --- | --- | --- | --- | --- |
|  |  | prevalence = 2% | prevalence = 10% | prevalence = 35% |
| New York inpatient [[6](#_ENREF_6)] | 4.3E-05 | 2.1E-03 | 4.3E-04 | 1.2E-04 |
| New York outpatient [[6](#_ENREF_6)] | 2.3E-06 | 1.2E-04 | 2.3E-05 | 6.6E-06 |
| Barcelona outpatient [[26](#_ENREF_26)] | 1.0E-04 | 5.0E-03 | **1.0E-03 ^a^** | 2.9E-04 |
| Barcelona inpatient [[26](#_ENREF_26)] | 2.9E-06 | 1.4E-04 | **2.9E-05 ^a^** | 8.2E-06 |
| ^a^ This is the best estimate used in the base case analysis. The Barcelona data was chosen because it is more recent and was collected from a network of treatment facilities that provided immigrant-specific incidence data in comparison to the New York study, which relied on Medicaid data and assumptions that all infected persons were immigrants. | | | | |

## 3. Comprehensive exam costs for intestinal parasite screening

*Health department costs*

CDC has developed a set of 12 guidelines for the comprehensive medical exam that is recommended for refugees post-resettlement, including a guideline for intestinal parasites [[39](#_ENREF_39)]. Among the remaining eleven guidelines, there is 1 general overview, 1 for conducting a physical examination and for collecting medical histories and 9 with specific information on other conditions and diseases.

We assumed that the cost of the comprehensive exam could be subdivided such that 10% of the activities would be devoted to intestinal parasites and 90% to other health conditions in refugees. This assumption is for the cost of screening in the absence of overseas presumptive treatment with either albendazole or ivermectin. The total health department cost would include a fraction of the comprehensive exam costs plus diagnostics (2 stool ova and parasites tests for hookworm, *Trichuris*, and *Ascaris* and 1 *Strongyloides* antibody test). Cost estimates were based on public and private reimbursement rates. The public reimbursement rate was estimated from the 2013 Centers for Medicare and Medicaid (CMS) Clinical Laboratory Fee Schedule and the 2013 CMS Physician Fee Schedule [[3](#_ENREF_3), [30](#_ENREF_30)]. The private reimbursement rates were summarized from the 2013 Physician’s Fee and Coding Guide [[31](#_ENREF_31)]. We assumed that health department costs for performing the exam and screening would be closer to the public reimbursement rate than to the private reimbursement rates. We used a weighted average of 25% x Physician’s Fee and Coding midpoint + 75% x CMS reimbursement rate to estimate the cost of screening (Table S5). The upper bound estimate was based on the high end of the reported reimbursement rates in the Physician Fee and Coding Guide. The lower bound was based on the CMS reimbursement rates. Medicare reimbursement rates were chosen instead of Medicaid reimbursement rates because there is less state-to-state variability.

We assumed that all refugees would require language translation assistance. Further, we assumed that 15 minutes of the translator’s time would be devoted to intestinal parasites. The cost of the translator was estimated using 2013 Bureau of Labor Statistics mean hourly wage data for translators, (occupation code 27-3091) $23.04 [[32](#_ENREF_32)]. In addition, we assumed that non-wage benefits would add 33% to hourly wages so that the total cost for 15 minutes of translator time would be 15 minutes /60 minutes per hour x $23.04 hourly wage x 1.33 adjustment for non-wage benefits = $7.66.

Table S5. Health department cost for screening for intestinal parasites assuming there are no overseas presumptive treatment programs (2013 USD)

| Source |  |  | Physician’s Fee and Coding Guide [[31](#_ENREF_31)] | | | CMS 2013 Clinical Lab Fee Schedule or Physician Fee Schedule [[3](#_ENREF_3), [30](#_ENREF_30)] |  |
| --- | --- | --- | --- | --- | --- | --- | --- |
| Outpatient costs | ICD-9 code | No. of tests or visits | Min (USD) | Max (USD) | Midpoint (USD) | National Midpoint or Facility Price (USD) | Total cost (weighted average)  (USD) |
| Comprehensive exam (new patient, level 4 complexity) | 99204 | 0.10 | 235 | 313 | 274 | 128.27 | 16.47 |
| Stool ova and parasites x2 | 87177 | 2 | 50 | 66 | 58 | 16.53 | 53.80 |
| Strongylides (Helminth antibody) | 86682 | 1 | 69 | 90 | 79.5 | 24.16 | 38.00 |
| Interpreter (0.25 hr) | 7.66 | | | | | | |
| Total |  |  | 200 | 261 | 231 | 78 | 116 |
| ^a^ Note that total costs are estimated using 25% * Physicians’ Fee and Coding Guide + 75% * CMS rate and are rounded to the nearest dollar. | | | | | | | |

If albendazole was provided during overseas presumptive treatment programs, the health department would only have to screen for *Strongyloides*. For this branch of the decision tree, we assumed that intestinal parasite screening would only comprise 5% of the comprehensive exam cost. In addition, we assumed that stool ova and parasites testing would be unnecessary; thus, the health department would only use the *Strongyloides* antibody test (Table S6).

Table S6. Health department cost for screening for *Strongyloides* assuming overseas presumptive treatment with albendazole (2013 USD)

| Source |  |  | Physician’s Fee and Coding Guide [[31](#_ENREF_31)] | | | CMS 2013 Clinical Lab Fee Schedule or Physician Fee Schedule [[3](#_ENREF_3), [30](#_ENREF_30)] |  |
| --- | --- | --- | --- | --- | --- | --- | --- |
| Outpatient costs | ICD-9 code | No. of tests or visits | Min (USD) | Max (USD) | Mean (USD) | National Midpoint or Facility Price (USD) | Total cost (weighted average) (USD) |
| Comprehensive exam (new patient, level 4 complexity) | 99204 | 0.05 | 235 | 313 | 274 | 128 | 8 |
| Strongylides (Helminth antibody) | 86682 | 1.00 | 69 | 90 | 79.5 | 24 | 38.00 |
| Interpreter (0.25 hr *$24.33/hr) | 7.66 | | | | | | |
| Total ^a^ |  |  | 88 | 113 | 101 | 38 | 54 |
| ^a^ Note that total costs are estimated using 25% * Physicians’ Fee and Coding Guide + 75% * CMS rate and are rounded to the nearest dollar. | | | | | | | |

If any intestinal parasite were diagnosed, we assumed that infected refugees would return for a follow-up outpatient visit (complexity level 2) and that the refugee would receive either albendazole or ivermectin for treatment. These treatment costs are summarized in Table S7. Drug costs were taken from the Red Book database and we assumed that, on average, treatment would require 2 x 200mg albendazole for hookworm, *Trichuris*, or Ascaris infections or 6 x 3mg ivermectin for *Strongyloides* infections. [[33](#_ENREF_33)]

Table S7. Treatment costs for refugees diagnosed with intestinal parasites during comprehensive exams in the United States after arrival (2013 USD)

| Source |  |  | Physician’s Fee and Coding Guide [[31](#_ENREF_31)] | | | CMS 2013 Clinical Lab Fee Schedule or Physician Fee Schedule [[3](#_ENREF_3), [30](#_ENREF_30)] |  |
| --- | --- | --- | --- | --- | --- | --- | --- |
| Outpatient costs | ICD-9 code | No. of tests or visits | Min (USD) | Max (USD) | Mean (USD) | National Midpoint or Facility Price (USD) | Total cost (weighted average) (USD) |
| Outpatient visit (existing patient, level 2 complexity) | 99212 | 1 | 92 | 125 | 108.5 | 24.50 | 45.50 |
| Interpreter (0.25 hr *$24.33/hr) | 7.66 | | | | | | |
| Albendazole (2 x 200 mg) | 119.40 | | | | | | |
| Ivermectin (6 x 3mg) | 33.50 | | | | | | |
| Total for hookworm, *Ascaris*, or *Trichuris* |  |  | 219 | 252 | 236 | 152 | 173 |
| Total for *Strongyloides* ^a^ |  |  | 133 | 166 | 150 | 66 | 87 |
| ^a^ Note that total costs are estimated using 25% * Physicians’ Fee and Coding Guide + 75% * CMS rate and are rounded to the nearest dollar. | | | | | | | |

*Patient opportunity and travel costs*

Patient opportunity costs were estimated based on assumed time requirements for patients to: undergo comprehensive exams, provide stool samples, and travel to and from appointments (Table S8). We estimated that it would require 2 hours for the comprehensive examination and 1 hour each to provide stool samples and/or to return for a follow-up visit and diagnosis. The time requirement for comprehensive exams was prorated as discussed above. The value of time was estimated using two sources: 1) the best and minimum cost estimates was the average GDP per capita-hour taken from International Monetary Fund data ($5.84 per hour) [[35](#_ENREF_35)] and 2) the maximum cost estimate was the average wage estimate across all occupations from the Bureau of Labor Statistics $22.33 [[32](#_ENREF_32)]. We believe the average GDP per capita-hour is the better estimate because many refugees may not be employed at the time of the initial comprehensive exam.

Table S8. Opportunity cost estimates for refugees participating in domestic screening programs (2013 USD)

| Screening for hookworm, *Ascaris*, *Trichuris*, and *Strongyloides* | | | | |  |  |
| --- | --- | --- | --- | --- | --- | --- |
|  | Time required (hours) | Fraction of exam spent on intestinal parasites | Average GDP per capita-hour [[35](#_ENREF_35)] | Average wage per hour [[32](#_ENREF_32)] | Min cost estimate | Max cost estimate |
| Comprehensive exam | 2 | 0.1 | 5.84 | 22.33 | 1.2 | 4.5 |
| Time to provide two stool samples | 0.5 | 2 | 5.84 | 22.33 | 5.84 | 22.33 |
| Total time cost | |  |  |  | 7.0 | 26.8 |
|  | | | | | | |
| Screening for *Strongyloides* only | | | | |  |  |
|  | Time required (hours) | No. of visits | Average GDP per capita-hour | Average wage per hour | Min cost estimate | Max cost estimate |
| Comprehensive exam | 2 | 0.05 | 5.84 | 22.33 | 0.58 | 2.23 |
| Total time cost | |  |  |  | 0.58 | 2.23 |
|  | | | | | | |
| Time required for follow visit after intestinal parasites diagnosis | | | | |  |  |
| Visit cost | 1 | 1 | 5.84 | 22.33 | 5.84 | 22.33 |

## 4. Outpatient and inpatient treatment cost estimates

For outpatient treatment, we assumed that doctors would run a battery of tests to identify the underlying illness. These tests are summarized in Table S9 below with the number of each test and cost estimates. The majority of patients with strongyloidiasis (range of 54-64%) in two previous studies were identified in patients referred for eosinophilia [[2](#_ENREF_2), [26](#_ENREF_26)]. Therefore, we assumed that outpatient visits might be coded as level 2 indicating that a low level of care outside of the diagnostic tests would be required. We assumed that two visits would be required. The first visit would include the initial testing, while the second would include diagnosis and prescription of medication.

Table S9. Strongyloidiasis outpatient cost estimates (2013 USD)

| Data source | | | | Physician’s Fee and Coding Guide [[31](#_ENREF_31)] | | | | CMS 2013 Clinical Lab Fee Schedule or Physician Fee Schedule [[3](#_ENREF_3), [30](#_ENREF_30)] |  |
| --- | --- | --- | --- | --- | --- | --- | --- | --- | --- |
| Outpatient costs | ICD-9 code | No. of visits/ tests | | Min | | Max | Midpoint | National Midpoint or Facility Price | Total cost (weighted average) |
| Level 2 outpatient visit x2 | 99212 | 2.00 | | 92.00 | | 125.00 | 108.50 | 24.50 | 91.00 |
| Stool ova and parasites x3 | 87177 | 3.00 | | 50.00 | | 66.00 | 58.00 | 16.53 | 80.69 |
| *Strongyloides* and Schistosomiasis (Helminth antibody) x2 | 86682 | 2.00 | | 69.00 | | 90.00 | 79.50 | 24.16 | 75.99 |
| CBC w differential | 85025 | 1.00 | | 38.00 | | 51.00 | 44.50 | 14.45 | 21.96 |
| Urinalysis | 81001 | 1.00 | | 29.00 | | 41.00 | 35.00 | 4.03 | 11.77 |
| Hepatic function panel | 80076 | 1.00 | | 50.00 | | 67.00 | 58.50 | 15.17 | 26.00 |
| Giardia antibody | 87329 | 1.00 | | 59.00 | | 77.00 | 68.00 | 22.28 | 33.71 |
| Cryptosporidium antibody | 87272 | 1.00 | | 59.00 | | 78.00 | 68.50 | 22.28 | 33.84 |
| Total ^a^ |  |  | | 683.00 | | 909.00 | 796.00 | 225.12 | 374.97 |
|  | | | | | | | | | |
| Albendazole cost (hookworm, trichuriasis or ascariasis diagnosis) | | | | | | | | | 119.40 |
| Ivermectin cost (strongyloidiasis diagnosis) | | | | | | | | | 33.50 |
|  | | | | | | | | | |
| Total + Albendazole ^a^ | | | 802.40 | | 1,028.40 | | 915.40 | 344.52 | 494.37 |
| Total + Ivermectin ^a^ | | | 716.50 | | 942.50 | | 829.50 | 258.62 | 408.46 |
| ^a^ Note that total costs are estimated using 25% * Physicians’ Fee and Coding Guide + 75% * CMS rate and are rounded to the nearest dollar. | | | | | | | | | |

*Strongyloidiasis hospitalization*

Cases requiring hospitalization are more expensive than those treated on an outpatient basis. As summarized in Table S10, data from the National Inpatient Survey collected by the Agency for Healthcare Research and Quality were used to estimate the cost of hospitalization [[34](#_ENREF_34)]. Annual data for 2005-2011 were extracted and updated to 2013 USD by using the medical consumer price index from the Bureau of Labor Statistics [[40](#_ENREF_40)].

Table S10. Average duration of stay, costs, and charges for hospitalized tuberculosis cases in the United States [[34](#_ENREF_34)]

| Year | No. of discharges | Mean length of stay | Charges in 2013 USD^a^ | Costs in 2013 USD^a^ |
| --- | --- | --- | --- | --- |
| 2011 | 94 | 10.7 | 97,937 | 24,107 |
| 2010 | 99 | 8.8 | 52,072 | 15,066 |
| 2009 | 98 | 13.0 | N/A | 24,023 |
| 2008 | 78 | 7.7 | 64,901 | 20,429 |
| 2007 | 84 | N/A | N/A | N/A |
| 2006 | 103 | N/A | N/A | N/A |
| 2005 | 73 | 7.7 | N/A | N/A |
| Case-weighted average ^b^ | | 9.7 | 57,714 | 19,776 |
| N/A: Data not available  ^a^ Costs and charged were adjusted to 2013 USD by using the U.S. Medical Consumer Price Index [[40](#_ENREF_40)]  ^b^ The case-weighted average was calculated by multiplying the annual means by the number of observations for a given year and then dividing by the total number of observations across all years. | | | | |

*Patient opportunity costs in the United States*

Patient opportunity costs were estimated using: 1) the average GDP per capita-hour ($5.84 per hour for 24 hours per day) [[35](#_ENREF_35)] for the minimum and best cost estimate and 2) the maximum cost estimate was based on the average wage estimate across all occupations $22.33 [[32](#_ENREF_32)] for 8-hour work days). We assumed that outpatient treatment would be associated with the loss of one full day for any of the intestinal parasites. We assumed that the opportunity cost for hospitalization could be estimated from the average duration of inpatient treatment for strongyloidiasis patients (rounded to 10 days) based on data from the National Inpatient Survey (Table S6). These estimates are summarized in Table S11.

Table S11. Estimated opportunity costs for outpatient and inpatient tuberculosis cases

|  | Opportunity costs | |  |
| --- | --- | --- | --- |
|  | Base | Min | Max |
| Outpatient opportunity cost (days) | 1 | | |
| Outpatient opportunity cost (USD) | 140 | 140 | 176 |
| Inpatient opportunity cost (days) | 10 | | |
| Inpatient opportunity cost (USD) | 1,400 | 1,400 | 1,760 |

## 5. Overseas presumptive treatment cost estimates

IOM provided 2013 data on costs from three sites in Asia: Thailand, Malaysia, and Nepal. The estimates from Thailand and Nepal were camp-based while those from Malaysia were for urban-based refugees. The cost estimates included 1) staff time (nurses, lab technicians, data entry, and support staff), 2) drug costs (albendazole 400mg, and ivermectin (18 mg), 3) pregnancy tests, 4) transportation costs, and 5) IOM overhead costs.

The costs by site are summarized in Table S12. The average cost per refugee is estimated by dividing total costs across all three sites by the total number of refugees treated across the three sites.

Table S12. Summary of cost data provided by IOM, 2013 USD

| **Budget Line** | **Thailand** | **Nepal** | | **Malaysia** | | **Total** |
| --- | --- | --- | --- | --- | --- | --- |
| Caseload | *5,500* | *10,000* | | *9,000* | | *24,500* |
| Staff & Office Cost | $ 33,629 | $ 51,385 | | $ 22,282 | | $ 107,296 |
| Operational Cost | $ 52,632 | $ 59,902 | | $ 33,480 | | $ 146,014 |
| Total | $ 90,574 | $ 116,851 | | $ 58,550 | | $ 269,975 |
|  | | | | | | |
| *Total cost per person (albendazole and ivermectin)* | *$ 16.47* | *$ 11.69* | | *$ 6.51* | | *$ 10.86* |
|  | | | | | | |
| Cost per drug, overseas treatment | Refugee-weighted mean | | Lower bound | | Upper bound | |
| Albenazole | $ 3.2 | | $ 2.7 | | $ 3.7 | |
| Ivermectin | $ 7.6 | | $ 3.8 | | $ 13.3 | |

## 6. Probability distributions

We used three types of probability distributions: beta, gamma, and uniform. The beta distribution was commonly used for probabilities that should be restricted to values between 0 and 1 (e.g., sensitivity, specificity, efficacy). The gamma distribution was used for positive real numbers (e.g., screening/treatment costs, outpatient/inpatient costs). The uniform distribution was used for parameters that were mostly based on expert opinion (e.g., QALY weights). For the beta and gamma distributions, parameters were estimated using the following formulas: **Mean** = (*Min* + 4**Best* + *Max*)/6, **Standard Deviation** = (*Max* – *Min*)/6. For these estimates, *Best* is our best estimate, *Max* is our maximum or upper bound estimate and *Min* is our minimum or lower bound estimate. We used Treeage software to fit distributions using the mean and standard deviations as reported in the Table S13.

Table S13. Estimated means and standard deviations for each uncertain parameter included in the analysis

| Description | Base case analysis | Lower bound | Upper bound | Distri-bution^a^ | Mean | Standard Deviation |
| --- | --- | --- | --- | --- | --- | --- |
| ***Epidemiological parameters*** | | | | | | |
| Baseline infection prevalence (without treatment) | | | | | | |
| Hookworm | 0.028 | 0.028 | 0.11 | B | 0.0690 | 0.0006 |
| *Ascaris* | 0.0084 | 0.0084 | 0.017 | B | 0.0127 | 0.0000 |
| *Trichuris* | 0.0056 | 0.0056 | 0.015 | B | 0.0103 | 0.0000 |
| *Strongyloides* | 0.2 | 0.06 | 0.34 | B | 0.2000 | 0.0467 |
| *Drug efficacy* | | | | | | |
| Albendazole against hookworm | 0.72 | 0.72 | 0.93 | B | 0.7550 | 0.0350 |
| Albendazole against *Trichuris* | 0.28 | 0.28 | 0.73 | B | 0.3550 | 0.0750 |
| Albendazole against *Ascaris* | 0.88 | 0.88 | 0.94 | B | 0.8900 | 0.0100 |
| Ivermectin against *Strongyloides* | 0.9 | 0.57 | 0.99 | B | 0.8600 | 0.0700 |
| *Test sensitivity* | | | | | | |
| Sensitivity of *Strongyloides* serologic test | 0.91 | 0.89 | 0.92 | B | 0.9083 | 0.0050 |
| Sensitivity of two stool O&P for hookworm | 0.78 | 0.53 | 0.88 | B | 0.8950 | 0.0150 |
| Sensitivity of two stool O&P for *Ascaris* | 0.81 | 0.57 | 0.95 | B | 0.9583 | 0.0083 |
| Sensitivity of two stool O&P for *Trichuris* | 0.96 | 0.81 | 0.99 | B | 0.9917 | 0.0017 |
| *Test specificity* | | | | | | |
| *Strongyloides* serologic test | 0.92 | 0.89 | 0.97 | B | 0.9233 | 0.0133 |
| Two stool O&P for hookworm*,* *Ascaris*, and *Trichuris* | 1 | 1 | 1 | NA |  |  |
| *Duration of risk of infection for outpatient illness* | | | | | | |
| hookworm | 6 | 5 | 7 | G | 6.0000 | 0.3333 |
| *Trichuris* | 2 | 1 | 2 | G | 1.8333 | 0.1667 |
| *Ascaris* | 1 | 1 | 1 | N/A |  |  |
| *Annual probability of seeking treatment given infection* | | | | | | |
| Outpatient visit for hookworm/ trichuriasis/ascariasis | 0.001 | 0.00012 | 0.005 | B | 0.0015 | 0.0008 |
| Outpatient visit for strongyloidiasis | 0.001 | 0.00012 | 0.005 | B | 0.0015 | 0.0008 |
| Inpatient strongyloidiasis | 2.90E-05 | 6.60E-06 | 1.20E-04 | B | 0.0000 | 0.0000 |
| Case fatality rate for inpatient strongyloidiasis | 0.167 | 0.02 | 0.25 | B | 0.1563 | 0.0383 |
| *Program parameters* | | | | | | |
| Proportion of refugees that present for comprehensive exam in United States | 0.9 | 0.8 | 1 | U | 0.9000 | 0.0033 |
| Probability that refugees will arrive from IOM facilities | 1 | 0.75 | 1 | U | 0.8500 | 0.0033 |
| Probability that refugee will receive presumptive treatment | 0.9 | 0.8 | 0.98 | U | 0.8900 | 0.0027 |
| Probability that stool O&P will be ordered given presumptive treatment | 0.05 | 0.03 | 0.07 | U | 0.0500 | 0.0001 |
| Adjustment factor for overseas versus domestic treatment 0-1 | 1 | 0.75 | 1 | U | 0.9583 | 0.0417 |
| *U.S. cost estimates (2013 USD)* | | | | | | |
| Screen for all nematodes | 116 | 78 | 260 | G | 133.7 | 30.3 |
| Screen for strongyloides, assuming albendazole presumptive treatment | 54 | 38 | 112 | G | 61.0 | 12.3 |
| Screen, assuming albendazole + ivermectin presumptive treatment | 78 | 57 | 171 | G | 90.0 | 19.0 |
| Albendazole treatment 400mg | 173 | 152 | 252 | G | 182.7 | 16.7 |
| Ivermectin treatment 18mg | 87 | 66 | 166 | G | 96.7 | 16.7 |
| Outpatient treatment for intestinal parasites (screening + albendazole cost) | 494 | 345 | 1030 | G | 553.8 | 114.2 |
| Outpatient treatment for intestinal parasites (screening + ivermectin cost) | 408 | 259 | 940 | G | 467.3 | 113.3 |
| Inpatient treatment for strongyloides | 20,000 | 15,000 | 24,000 | G | 19833.3 | 1500.0 |
| *Overseas cost estimates (2013 USD) of presumptive treatment in Asia* | | | | | | |
| Albendazole | 3.2 | 2.7 | 3.7 | G | 3.95 | 0.45 |
| Ivermectin | 7.6 | 3.8 | 13.3 | G | 8.85 | 1.58 |
| *Opportunity cost estimates (2013 USD)* | | | | | | |
| Screening (all parasites) | 7 | 7 | 22 | G | 9.5 | 2.5 |
| Screening (*Strongyloides* only) | 1 | 1 | 2.2 | G | 1.2 | 0.2 |
| Treatment after screening | 6 | 6 | 22 | G | 8.67 | 2.67 |
| Outpatient cases | 140 | 140 | 176 | G | 146 | 6 |
| Inpatient strongyloides | 1,400 | 1,400 | 1,760 | G | 1460 | 60 |
| *QALY estimates* | | | | | | |
| QALY decrement for *Strongyloides* infections | 0.001 | 0 | 0.01 | B | 0.0023 | 0.0017 |
| QALY decrement for hookworm, *Ascaris*, *Trichuris* infections | 0.001 | 0 | 0.01 | B | 0.0023 | 0.0017 |
| ^a^ Distribution types: U- uniform, B- beta, G- gamma | | | | | | |

## 7. Baseline disease burden by parasite

The baseline disease burden by parasite was estimated using the model. For each outcome measure (cases, hospitalizations, deaths, life years, and QALYs), we calculate the total number of each measure for by setting prevalence rates for three parasites to zero to isolate the burden for the remaining parasite. Then, we calculated the number of each outcome measure. The results are summarized in Table S14.

TableS14. Breakdown of cases, hospitalizations, deaths, life years and QALYs by causative parasite (60-year time horizon for a one-year cohort of 27,700 Asian refugees) ^a^

|  | *Hookworm, Trichuris, and Ascaris* | *Strongyloides* |
| --- | --- | --- |
| Cases | 6.0 | 138 |
| Hospitalizations | 0 | 4.0 |
| QALYs | 5.5 | 148 |
| LY | 0 | 13 |
| deaths | 0 | 0.67 |

^a^ Results are rounded

## 8. Additional sensitivity analysis

In addition to the sensitivity analysis presented in the main article, we performed additional sensitivity analysis using data from the Monte Carlo Simulation analysis (10,000 iterations). Table S15 shows the range of estimates for the incremental cost per QALY for three pairs of alternatives: 1) ‘Domestic Screening and Treatment’ compared to “No Program”, 2) ‘Domestic Screening and Treatment’ compared to “Overseas Albendazole and Ivermectin”, and 3) “Overseas Albendazole and Ivermectin” compared to “No Program”. Relative to “No Program”, the “Overseas Albendazole and Ivermectin” program has an almost 90% probability of costing less than $14,000 per QALY gained and a greater than 99% probability of costing less than $50,000 per QALY gained. In comparison, “Domestic Screening and Treatment” is much more costly per QALY gained relative to “No Program”. There is a 16% probability that the cost per QALY gained will be greater than $50,000 and a 75% probability it would cost more than $13,000. Comparing “Domestic Screening and Treatment” to “Overseas Albendazole and Ivermectin”, there is a 65% probability that the overseas treatment program would be both more effective and less costly than domestic screening. Even when domestic screening is more effective, there is only about a 1% probability that that the cost per QALY would be less than $50,000 compared to overseas presumptive treatment.

Table S15. Percentile estimates of the cost per QALY gained, 10,000 iterations in 2013 USD

| “Domestic Screening and Treatment” vs. “No Program” | |  | “Domestic Screening and Treatment” vs. “Overseas Albendazole and Ivermectin” | |  | “Overseas Albendazole and Ivermectin” vs. “No Program” | |
| --- | --- | --- | --- | --- | --- | --- | --- |
| 1 percentile | 4,815 |  | 1 percentile | (12,075,242) |  | 1 percentile | 458 |
| 2.5 percentile | 6,029 |  | 2.5 percentile | (5,196,397) |  | 2.5 percentile | 600 |
| 5 percentile | 7,421 |  | 5 percentile | (2,628,153) |  | 5 percentile | 772 |
| 10 percentile | 8,732 |  | 10 percentile | (1,221,610) |  | 10 percentile | 1,070 |
| 25 percentile | 13,156 |  | 25 percentile | (401,321) |  | 25 percentile | 1,912 |
| 50 percentile | 21,870 |  | 50 percentile | (145,656) |  | 50 percentile | 3,446 |
| 75 percentile | 39,852 |  | 75 percentile | 128,081 |  | 75 percentile | 7,024 |
| 90 percentile | 77,041 |  | 90 percentile | 877,380 |  | 90 percentile | 13,847 |
| 95 percentile | 114,246 |  | 95 percentile | 1,850,790 |  | 95 percentile | 20,580 |
| 97.5 percentile | 168,451 |  | 97.5 percentile | 4,130,060 |  | 97.5 percentile | 29,510 |
| 99 percentile | 268,034 |  | 99 percentile | 8,882,370 |  | 99 percentile | 50,210 |

Scatter plots of the incremental cost and incremental effectiveness per person are shown in Figure S4a-c for the first 1000 iterations of the Monte Carlo Simulation analysis. Each graph also shows three lines for which the cost per QALY gained is 1) $10,000, 2) $25,000, and 3) $50,000. As effectiveness increases, the cost of the program increases along the line to maintain a threshold value (e.g., an incremental effectiveness of 0.001 QALYs and increment cost of $10 has the same cost per QALY ratio as that for an incremental effectiveness of 0.002 QALYs and an incremental cost of $20). Each point located to the right of the lines would have a cost per QALY gained less than the thresholds. Points to the left of the lines have a cost per QALY gained greater than the thresholds.

Figure S4a shows that the incremental cost per person of “Domestic Screening and Treatment” compared to “No Program” is usually in the range of $100-$200 and that the incremental effectiveness per person is usually between 0 and 0.02 QALYs per person. The majority of points are to the left of the $50,000 per QALY threshold and to the right of the $10,000 per QALY threshold. Figure S4b shows that the incremental cost per person of “Domestic Screening and Treatment” compared to “Overseas Albendazole and Ivermectin” is usually in the range of $70-$200 and that the incremental effectiveness per person is usually between -0.02 and 0.02 QALYs per person. The majority of points are to the left of the $10,000 per QALY threshold and that effectiveness is more commonly better for “Overseas Albendazole and Ivermectin”. Figure S4c shows that the incremental cost per person of “Overseas Albendazole and Ivermectin” compared to “No Program” is usually in the range of $10-$40 and that the incremental effectiveness per person is usually between 0 and 0.02 QALYs per person. The majority of points are to the left of the $10,000 per QALY threshold and that “Overseas Albendazole and Ivermectin” is never cost saving.

Figure S4a. Scatter plot of incremental cost and incremental effectiveness, “Domestic Screening and Treatment” vs. “No Program”

Figure S4b. Scatter plot of incremental cost and incremental effectiveness, “Domestic Screening and Treatment” vs. “Overseas Albendazole and Ivermectin”

Figure S4c. Scatter plot of incremental cost and incremental effectiveness, “Overseas Albendazole and Ivermectin” vs. “No Program”

## 8. Two Additional Alternatives: Domestic Albendazole + Ivermectin and Domestic Albendazole and Screening for *Strongyloides*

This section provides analyses for two additional alternatives: “Domestic Albendazole and Ivermectin” and “Domestic Albendazole and Screening for *Strongyloides*”. These alternatives are recommended in CDC guidelines [[41](#_ENREF_41)] for refugees arriving from countries without presumptive treatment programs. These programs are very similar to the options presented in the article: “Overseas Albendazole and Ivermectin” and “Overseas Albendazole and Domestic Screening for *Strongyloides*”. The only difference is that presumptive treatment is provided after arrival in the United States rather than prior to departure for the United States. These alternatives were not included in the article because drug prices are much higher in the United States and because we do not anticipate any major differences in health outcomes. The cost for “Domestic Albendazole and Ivermectin” is about $165 per refugee as summarized in Table S16. This is much greater than the estimated cost overseas ($10.80). Because of the big difference in costs between domestic and overseas presumptive treatment, this alternative is only included in the appendix.

The cost of domestic presumptive treatment with albendazole only is included in Table S17. This cost can be added to the cost of domestic screening for *Strongyloides* infection (Table S6) to estimate the total program cost for “Domestic Albendazole and Screening for *Strongyloides*”. The total cost will also include follow-up treatment with ivermectin for individuals who test positive (Table S7).

Table S16. Cost estimates for “Domestic Albendazole and Ivermectin” in 2013 USD

| Source |  |  | Physician’s Fee and Coding Guide [[31](#_ENREF_31)] | | | CMS 2013 Clinical Physician Fee Schedule [[3](#_ENREF_3)] |  |
| --- | --- | --- | --- | --- | --- | --- | --- |
| Outpatient costs | ICD-9 code | No. of tests or visits | Min (USD) | Max (USD) | Midpoint (USD) | National Midpoint or Facility Price (USD) | Total cost (weighted average) |
|  |  |  |  |  |  |  | (USD) |
| Comprehensive exam (new patient, level 4 complexity) | 99204 | $0.05 | $235 | $313 | $274 | $128.27 | $8.24 |
| Albendazole drug cost | NA | NA | NA | NA | NA | NA | $119.40 |
| Ivermectin | NA | NA | NA | NA | NA | NA | $33.50 |
| Interpreter (0.125 hr) | 3.83 | | | | | | |
| Total |  |  | $168.48 | $172.38 | $170.43 | $163.14 | $164.96 |
| ^a^ Note that total costs are estimated using 25% * Physicians’ Fee and Coding Guide + 75% * CMS rate and are rounded to the nearest dollar. | | | | | | | |

Table S17. Cost estimates for domestic presumptive treatment with albendazole only, 2013 USD

| Source |  |  | [Physician’s Fee and Coding Guide [[31](#_ENREF_31)]](file:///C:\Users\wqm7\AppData\Local\Microsoft\Windows\Temporary%20Internet%20Files\Content.Outlook\3P6IX51V\Copy%20of%20Copy%20of%20Costing%20overseas%20r9.xlsx#RANGE!_ENREF_32) | | | CMS 2013 Clinical Lab Fee Schedule or Physician Fee Schedule [[3](#_ENREF_3)] |  |
| --- | --- | --- | --- | --- | --- | --- | --- |
| Outpatient costs | ICD-9 code | No. of tests or visits | Min (USD) | Max (USD) | Midpoint (USD) | National Midpoint or Facility Price (USD) | Total cost (weighted average) |
|  |  |  |  |  |  |  | (USD) |
| Comprehensive exam (new patient, level 4 complexity) | 99204 | 0.05 | $235 | $313 | $274 | $128.27 | $ 8.24 |
| Albendazole drug cost | NA | NA | NA | NA | NA | NA | $ 119.40 |
| Ivermectin | NA | NA | NA | NA | NA | NA | 0 |
| Interpreter (0.125 hr) | 3.83 | | | | | | |
| Total |  |  |  |  |  |  | $ 131.47 |
| ^a^ Note that total costs are estimated using 25% * Physicians’ Fee and Coding Guide + 75% * CMS rate and are rounded to the nearest dollar. | | | | | | | |

The estimated costs and health outcomes of ‘Domestic Albendazole + Ivermectin” and’ Domestic Albendazole and Screening for *Strongyloides’* compared to other alternatives is shown in Table S18. The cost is much higher relative to overseas presumptive treatment programs and expected health outcomes are the same. Thus, these alternatives are not preferred because it costs approximately 10 times more to provide presumptive treatment in the United States compared to providing the same treatment regimen overseas prior to departure.

Table S18. Estimates costs and health outcomes of Domestic Albendazole and Ivermectin and Domestic Albendazole and Screening for *Strongyloides* compared to other alternatives

|  | No program' | “Overseas Albendazole and Ivermectin” | “Domestic Screening and Treatment' | “Overseas Albendazole and Domestic Screening for *Strongyloides”* | “Domestic Albendazole and Iverrmectin” | “Domestic Albendazole and Screening for *Strongyloides*” |
| --- | --- | --- | --- | --- | --- | --- |
| **Costs, 2013 USD** |  |  |  |  |  |  |
| **Total costs** | **$165,923** | **$418,824** | **$3,832,572** | **$2,182,483** | **$4,146,136** | **$5,227,821** |
|  |  |  |  |  |  |  |
| **Health outcomes (discounted)** | |  |  |  |  |  |
| Outpatient cases | 145 | 29 | 40 | 39 | 29 | 28 |
| Hospitalizations | 4.0 | 0.8 | 1.1 | 1.1 | 0.8 | 1.1 |
| Deaths | 0.67 | 0.13 | 0.18 | 0.18 | 0.13 | 0.18 |
| Life years | 700,526 | 700,536 | 700,535 | 700,535 | 700,536 | 700,535 |
| QALYs | 605,253 | 605,377 | 605,366 | 605,366 | 605,377 | 605,377 |
| Economic outcomes relative to “No Program”, 2013 USD | | |  |  |  |  |
| Net cost per case averted |  | $2,146 | $34,907 | $ 18,483 | $34,213 | $43,257 |
| Net cost per hospitalization averted | | $76,606 | $1,238,977 | $ 680,735 | $1,251,602 | $1,224,141 |
| Net cost per death averted |  | $458,718 | $7,419,026 | $4,076,259 | $7,330,188 | $10,246,183 |
| Net cost per life year gained | | $24,036 | $388,754 | $ 213,594 | $392,708 | $384,092 |
| Net cost per QALY gained |  | $2,219 | $32,706 | $18,167 | $2,750 | $24,872 |

## 9. Additional analyses for U.S.-bound refugees from Africa

An estimated 13,800 African refugees settle in the United States each year. [[4](#_ENREF_4)] The prevalence of hookworm, *Ascaris*, and *Trichuris* infections among African refugees is estimated using the results from Swanson et al. [[20](#_ENREF_20)] using the same approach as was used for these infections among refugees from Asia (see Appendix section 2). The resulting prevalence rates vary from 0.011 for hookworm to 0.069 for *Trichuris* (Table S19).

Table S19. Estimated prevalence rates for hookworm, *Ascaris*, and *Trichuris* infections among African refugees after adjustment for presumptive treatment efficacy

|  | Ascaris | Hookworm | Trichuris |
| --- | --- | --- | --- |
| Prevalence after treatment [[20](#_ENREF_20)] | 0.003 | 0.003 | 0.05 |
|  |  |  |  |
| Meta analysis efficacy [[15](#_ENREF_15)] | 0.88 | 0.72 | 0.28 |
| Adjusted pre-treatment prevalence based on meta-analysis efficacy | 0.025 | 0.011 | 0.069 |

*Strongyloides* prevalence was estimated using data from four refugee-specific studies that used serologic testing in the United States and Africa. Among the four studies, three studies reported prevalence rates between 31% and 42%. The fourth study showed a much lower prevalence rate (2%). The estimated prevalence was adjusted using sensitivity and specificity for *Strongyloides* serologic tests (Table S1) as described in Section 2 of this appendix. Overall, the prevalence was estimated to be 33% based on the sample-size weighted average prevalence from the four studies after adjusting for test sensitivity and specificity.

Table S20. Estimated prevalence rates for *Strongyloides* infections among African refugees after adjustment for test sensitivity and specificity

| Site | Sample size | Africa | | Country of Origin or Last Residence in Africa | Reference |
| --- | --- | --- | --- | --- | --- |
|  |  | Unadjusted  prevalence | Adjusted  pervalence |  |  |
| San Diego, CA 2006-07 | 172 | 33% | 30% | Sudan | Brodine et al. [[42](#_ENREF_42)] |
| Santa Clara, CA 2008-10 | 29 | 31% | 28% |  | Chang et al. [[22](#_ENREF_22)] |
| 2000-02 Melbourne Australia | 124 | 2% | 0% | East Africa | Caruana et al. [[21](#_ENREF_21)] |
| United States 2004 | 562 | 42% | 41% | Somalia and Sudan | Posey et al. [[43](#_ENREF_43)] |
| Total | 887 | 34% | 33% |  |  |

To be conservative the cost of overseas presumptive treatment was assumed to be 25% higher for African refugees compared to Asian refugees. IOM has provided 2013 data for the cost of overseas presumptive treatment for African refugees. Since the program in Africa also includes praziquantel for schistosomiasis and coartem for malaria, the per-refugee cost is higher in Africa ($33.6 vs. $10.8). However, the coartem regimen requires 6 doses over 3 days compared to 3 doses total over two days for both albendazole and ivermectin. The albendazole and ivermectin drug costs are similar in Africa and Asia. However, we decided to multiply the Asian costs ($3.20 for albendazole and $10.80 for albendazole and ivermectin) by 1.25 to be conservative.

Besides prevalence rates and overseas presumptive treatment costs, all other parameters were carried over from the analysis of Asian refugees (Table S1). The results for African refugees are similar to those for Asian refugees. The cost per QALY gained ($1,338) is lowest for “Overseas Albendazole and Ivermectin”. This is also the most effective option; other options are limited by the sensitivity of tests to determine helminth infection.

Table S21. Estimates costs and health outcomes of helminth screening and treatment alternatives for African refugees (n=13,800)

|  | “No program” | “Overseas Albendazole and Ivermectin” | “Domestic Screening and Treatment” | “Presumptive Albendazole + Domestic Screening for Strongyloides” | “Domestic Albendazole + Ivermectin” | “Domestic Albendazole and Screening for Strongyloides” |
| --- | --- | --- | --- | --- | --- | --- |
| **Costs, 2013 USD** |  |  |  |  |  |  |
| **Total costs** | **$273,676** | **$509,403** | **$4,382,971** | **$2,077,176** | **$2,736,264** | **$5,581,550** |
|  |  |  |  |  |  |  |
| **Health outcomes (discounted)** | |  |  |  |  |  |
| Outpatient cases | 239 | 50 | 65 | 66 | 66 | 65 |
| Hospitalizations | 6.6 | 1.3 | 1.7 | 1.7 | 1.7 | 1.7 |
| Deaths | 1.11 | 0.21 | 0.29 | 0.29 | 0 | 0 |
| Life years | 700,518 | 700,535 | 700,533 | 700,533 | 700,533 | 700,533 |
| QALYs | 605,154 | 605,356 | 605,338 | 605,339 | 605,339 | 605,338 |
| Economic outcomes relative to “No Program”, 2013 USD | | |  |  |  |  |
| Net cost per case averted |  | $1,264 | $23,593 | $12,410 | $23,224 | $31,111 |
| Net cost per hospitalization averted | | $76,606 | $44,667 | $843,390 | $453,454 | $798,728 |
| Net cost per death averted |  | $267,470 | $5,050,242 | $2,715,294 | $4,782,802 | $4,782,802 |
| Net cost per life year gained | | $24,036 | $14,016 | $264,647 | $142,289 | $250,633 |
| Net cost per QALY gained |  | $1,338 | $22,541 | $12,099 | $21,283 | $28,644 |

References

1. Martin DC, Yankay JE. Refugees and Asylees 2011. DHS, Office of Immigration Statistics Policy, 2012.

2. Milder J, Walzer P, Kilgore G, Rutherford I, Klein M. Clinical features of Strongyloides stercoralis infection in an endemic area of the United States. Gastroenterology. 1981;80:1481-4188.

3. Centers for Medicare and Medicaid Services. Physician Fee Schedule, <http://www.cms.gov/Medicare/Medicare-Fee-for-Service-Payment/PhysicianFeeSched/index.html>. accessed on March 20, 2013. 2013.

4. Department of Homeland Security (2012) Yearbook of Immigration Statistics <http://www.dhs.gov/yearbook-immigration-statistics-2011-0>, accessed on October 1, 2012. Washington, D.C.

5. Centers for Disease Control and Prevention, National Center for Health Statistics. Underlying Cause of Death 1999-2010 on CDC WONDER Online Database, released 2012. Data are from the Multiple Cause of Death Files, 1999-2010, as compiled from data provided by the 57 vital statistics jurisdictions through the Vital Statistics Cooperative Program. Accessed at <http://wonder.cdc.gov/ucd-icd10.html> on Jul 8, 2014

6. Muennig P, Pallin D, Challah C, Khan K. The cost-effectiveness of ivermectin vs. albendazole in the presumptive treatment of strongyloidiasis in immigrants to the United States. Epidemiology and Infection. 2004;132(6):1055-63.

7. Salomon JA, Vos T, Hogan DR, Gagnon M, Naghavi M, Mokdad PA, et al. Common values in assessing health outcomes from disease and injury: disability weights measurement study for the Global Burden of Disease Study 2010. The Lancet. 2012;380(9859):2129-43.

8. Krolewiecki AJ, Lammie P, Jacobson J, Gabrielli A-F, Levecke B, Socias E, et al. A Public Health Response against Strongyloides stercoralis : Time to Look at Soil-Transmitted Helminthiasis in Full. PLoS Neglected Tropical Diseases. 2013;7(5):e2165.

9. Requena-Mendez A, Chiodini P, Bisoffi Z, Buonfrate D, Gotuzzo E, Munoz J. The Laboratory Diagnosis and Follow Up of Strongyloidiasis: A Systematic Review. PLoS Neglected Tropical Diseases. 2013;7(1):e2002.

10. Tarafder M, Carabin H, Joseph L, Balolong Jr E, Olveda R, McGarvey S. Estimating the sensitivity and specificity of Kato-Katz stool examination technique for detection of hookworms, Ascaris lumbricoides and Trichuris trichiura infections in humans in the absence of a ‘gold standard’. Interanational Journal of Parasitology. 2010;40(4):399-404.

11. van Doorn HR, Koelewijn R, Hofwegen H, Gilis H, Wetsteyn JCFM, Wismans PJ, et al. Use of Enzyme-Linked Immunosorbent Assay and Dipstick Assay for Detection of Strongyloides stercoralis Infection in Humans. Journal of Clinical Microbiology. 2007;45(2):438-42.

12. Bisoffi Z, Buonfrate D, Sequi M, Mejia R, Cimino RO, Krolewiecki AJ, et al. Diagnostic Accuracy of Five Serologic Tests for Strongyloides stercoralis Infection. PLoS Neglected Tropical Diseases. 2014;8(1):e2640.

13. Nikolay B, Brooker SJ, Pullan RL. Sensitivity of diagnostic tests for human soil-transmitted helminth infections: a meta-analysis in the absence of a true gold standard. International Journal for Parasitology. 2014;44:765-74.

14. Cartwright CP. Utility of Multiple-Stool-Specimen Ova and Parasite Examinations in a High-Prevalence Setting. Journal of Clinical Microbiology. 1999;37(8):2408-11.

15. Keiser J, Utzinger J. Efficacy of Current Drugs Against Soil-Transmitted Helminth Infections Systematic Review and Meta-analysis. Journal of the American Medical Association. 2008;299(16):1937-48.

16. CDC. Parasites - Strongyloides. CDC Global Health - Division of Parasitic Diseases and Malaria, <http://www.cdc.gov/parasites/strongyloides/health_professionals/index.html> accessed on November 13, 2012, 2012.

17. Bisoffi Z, Buonfrate D, Angheben A, Boscolo M, Anselmi M, Marocco S, et al. Randomized Clinical Trial on Ivermectin versus Thiabendazole for the Treatment of Strongyloidiasis. PLoS Neglected Tropical Diseases. 2011;5(7):e1254.

18. Igual-Adell R, Oltra-Alcaraz C, Soler-Company E, Sánchez-Sánchez P, Matogo-Oyana J, Rodríguez-Calabuig D. Efficacy and safety of ivermectin and thiabendazole in the treatment of strongyloidiasis. Expert Opinion on Pharmatherapy. 2004;5(12):2615-9.

19. Suputtamongkol Y, Premasathian N, Bhumimuang K, Waywa D, Nilganuwong S, Karuphong E, et al. Efficacy and Safety of Single and Double Doses of Ivermectin versus 7-Day High Dose Albendazole for Chronic Strongyloidiasis. PLoS Neglected Tropical Diseases. 2011;5(5):e1044.

20. Swanson SJ, Phares CR, Mamo B, Smith KE, Cetron MS, Stauffer WM. Albendazole therapy and enteric parasites in United States-bound refugees. New England Journal of Medicine. 2012;366(16):1498-507.

21. Caruana SR, Kelly HA, Ngeow JYY, Ryan NJ, Bennett CM, Chea L, et al. Undiagnosed and Potentially Lethal Parasite Infections Among Immigrants and Refugees in Australia. Journal of Travel Medicine. 2006;13(4):233-9.

22. Chang A, Perry S, Du J, Agunbiade A, Polesky A, Parsonnet J. Decreasing intestinal parasites in recent northern California refugees. American Journal of Tropical Medicine and Hygiene. 2013;88(1):191-7.

23. Dawson-Hahn EE, Greenberg SLM, Domachowske JB, Olson BG. Eosinophilia and the Seroprevalence of Schistosomiasis and Strongyloidiasis in Newly Arrived Pediatric Refugees: An Examination of Centers for Disease Control and Prevention Screening Guidelines The Journal of Pediatrics. 2010;156(6):1016-8.e1.

24. Gyorkos TW, Genta RM, Viens P, MacLean JD. Seroepidemiology of Strongyloides infection in the Southeast Asian refugee population in Canada. American Journal of Epidemiology. 1990;132(2):257-64.

25. Paxton G, Sangster K, Maxwell E, CRJ M, Drewe R. Post-arrival health screening in Karen refugees in Australia. PLoS ONE. 2012;7(5):e38194.

26. Valerio L, Roure S, Fernandez-Rivas G, Basile L, Martınez-Cuevasa O, Ballesteros A-L, et al. Strongyloides stercoralis, the hidden worm. Epidemiological and clinical characteristics of 70 cases diagnosed in the North Metropolitan Area of Barcelona, Spain, 2003–2012. Transactions of the Royal Society of Tropical Medicine and Hygiene. 2013;107:465-70.

27. Muennig P, Pallin D, Sell RL, Chan M-S. The Cost Effectiveness of Strategies for the Treatment of Intestinal Parasites in Immigrants. New England Journal of Medicine. 1999;340(10):773-9.

28. Newberry AM, Williams DN, Stauffer WM, Boulware DR, Hendel-Paterson BR, Walker PF. Strongyloides Hyperinfection Presenting as Acute Respiratory Failure and Gram-Negative Sepsis. Chest 2005 November ; 128(5): 3681–3684. 2005;128(5):3681-4.

29. Mohammed KA, Haji HJ, Gabrielli A-F, Mubila L, Gautam Biswas, Chitsulo L, et al. Triple Co-Administration of Ivermectin, Albendazole and Praziquantel in Zanzibar: A Safety Study. PLoS Neglected Tropical Diseases. 2008;2(1):e171.

30. Centers for Medicare and Medicaid Services. Clinical Laboratory Fee Schedule, <http://www.cms.gov/Medicare/Medicare-Fee-for-Service-Payment/ClinicalLabFeeSched/clinlab.html>. Accessed on March 20, 2013. 2013.

31. InGauge Healthcare Solutions. 2013 Physicians' Fee & Coding Guide. Atlanta GA2013.

32. Bureau of Labor Statistics, 2013 National Occupational Employment and Wage Estimates. Washington DC, <http://www.bls.gov/oes/current/oes_nat.htm#29-0000>, Accessed on May 1, 2014.

33. Healthcare Series [Red Book]. Greenwood Village, Colo: Thomson Reuters (Healthcare) Inc. Updated periodically. Accessed on May 1, 2013 [Internet]. 2013.

34. HCUP Nationwide Inpatient Sample (NIS). Healthcare Cost and Utilization Project (HCUP). 2007-2009. Agency for Healthcare Research and Quality, Rockville, MD. [www.hcup-us.ahrq.gov/nisoverview.jsp](http://www.hcup-us.ahrq.gov/nisoverview.jsp)

35. 2013 World Economic Outlook database. <http://www.imf.org/external/pubs/ft/weo/2013/01/weodata/index.aspx> Accessed on June 3, 2013. [Internet]. 2013.

36. Bethony J, Brooker S, Albonico M, Geiger SM, Loukas A, Diemert D, et al. Soil-transmitted helminth infections: ascariasis, trichuriasis, and hookworm. The Lancet. 2006;367:1521-32.

37. Hanmer J, Lawrence WF, Anderson JP, Kaplan RM, Fryback DG. Report of Nationally Representative Values for the Noninstitutionalized US Adult Population for 7 Health-Related Quality-of-Life Scores. Med Decis Making. 2006;4:391-400.

38. Schär F, Trostdorf U, Giardina F, Khieu V, Muth S, Marti H, et al. Strongyloides stercoralis: Global Distribution and Risk Factors PLoS Neglected Tropical Diseases. 2013;7(7):e2288.

39. CDC. Refugee Health Guidelines: Intestinal Parasites Overseas Recommendations Recommendations for Overseas Presumptive Treatment of Intestinal Parasites for Refugees Destined for the United States. Atlanta, <http://www.cdc.gov/immigrantrefugeehealth/guidelines/overseas/intestinal-parasites-overseas.html>, accessed on October 1, 2012: 2012.

40. Consumer Price Index, Average Annual Indexes 2006-12, <http://www.bls.gov/cpi/>, accessed on May 1, 2013 [Internet]. 2013.

41. CDC. Guidelines for the U.S. Domestic Medical Examination for Newly Arriving Refugees <http://www.cdc.gov/immigrantrefugeehealth/guidelines/domestic/domestic-guidelines.html>, Accessed on May 5, 2014. 2014.

42. Brodine SK, Thomas A, Huang R, Harbertson J, Mehta S, Leake J, et al. Community Based Parasitic Screening and Treatment of Sudanese Refugees: Application and Assessment of Centers for Disease Control Guidelines. American Journal of Tropical Medicine and Hygiene. 2009;80(3):425-30.

43. Posey DL, Blackburn BG, Weinberg M, Flagg EW, Ortega L, Wilson M, et al. High Prevalence and Presumptive Treatment of Schistosomiasis and Strongyloidiasis among African Refugees. Clinical Infectious Diseases. 2007;45:1310-5.
