# Supplementary material for: Economic Analysis of the Impact of Overseas and Domestic Treatment and Screening Options for Intestinal Helminth Infection among US-Bound Refugees from Asia
Source: PLoS Negl Trop Dis. 2016 Aug 10;10(8):e0004910. doi: 10.1371/journal.pntd.0004910 (PMC4980012; doi:10.1371/journal.pntd.0004910)
Supplement: S1 Table — (DOCX) [file pntd.0004910.s002.docx]

Table S1. Epidemiological and economic input parameters

| Description | Base case analysis | Low | High | Distri-bution ^a^ | Ref |
| --- | --- | --- | --- | --- | --- |
| ***Epidemiological parameters*** | | | | | |
| Baseline infection prevalence (without treatment) ^b^ | | | | | |
| Hookworm | 0.028 | 0.028 | 0.11 | B | [1] |
| *Ascaris* | 0.0084 | 0.0084 | 0.017 | B | [1] |
| *Trichuris* | 0.0056 | 0.0056 | 0.015 | B | [1] |
| *Strongyloides* | 0.20 | 0.06 | 0.34 | B | [2-6] |
| *Drug efficacy* ^b^ |  |  |  |  |  |
| Albendazole against hookworm | 0.72 | 0.72 | 0.93 | B | [1, 7, 8] |
| Albendazole against *Trichuris* | 0.28 | 0.28 | 0.73 | B | [1, 7, 8] |
| Albendazole against *Ascaris* | 0.88 | 0.88 | 0.94 | B | [1, 7, 8] |
| Ivermectin against *Strongyloides* | 0.90 | 0.57 | 0.99 | B | [9-11] |
| *Test sensitivity* ^b^ | | | | | |
| *Strongyloides* serologic test | 0.91 | 0.89 | 0.92 | B | [12] |
| Two stool O&P for hookworm | 0.78 | 0.53 | 0.88 | B | [13, 14] |
| Two stool O&P for *Ascaris* | 0.81 | 0.57 | 0.95 | B | [13, 14] |
| Two stool O&P for *Trichuris* | 0.96 | 0.81 | 0.99 | B | [13, 14] |
| *Test specificity* ^b^ | | | | | |
| *Strongyloides* serologic test | 0.92 | 0.89 | 0.97 | B | [12, 15, 16] |
| Two stool O&P for hookworm, *Ascaris*, or *Trichuris* | 1 | 1 | 1 | NA | Assumption |
| *Duration of infection* ^b^ | | | | | |
| Hookworm | 6 | 5 | 7 | G | [17] |
| *Trichuris* | 2 | 1 | 2 | G | [17] |
| *Ascaris* | 1 | 1 | 1 | N/A | [17] |
| *Annual probability of seeking treatment given infection* ^b^ | | | | | |
| Outpatient visit for hookworm/ trichuriasis/ascariasis | 0.001 | 0.00012 | 0.005 | B | [18, 19] |
| Outpatient visit for strongyloidiasis | 0.001 | 0.00012 | 0.005 | B | [18, 19] |
| Inpatient strongyloidiasis | 2.9E-05 | 6.6E-06 | 1.2E-04 | B | [18, 19] |
| Case fatality rate for inpatient strongyloidiasis | 0.167 | 0.02 | 0.25 | B | [20] |
| *Program parameters* | | | | | |
| Proportion of refugees receiving domestic comprehensive medical exam | 0.9 | 0.8 | 1.0 | U | Assumption |
| Probability refugees arrive from countries with presumptive treatment program | 1.00 | 0.75 | 1.00 | U | IOM report |
| Probability refugees receive presumptive treatment | 0.90 | 0.80 | 0.98 | U | Assumption |
| Probability stool O&P will be ordered given presumptive treatment | 0.05 | 0.03 | 0.07 | U | Assumption |
| Adjustment factor for overseas versus domestic treatment 0-1 | 1 | 0.75 | 1 | U | Assumption |
| *Demographics* | | | | | |
| Probability of death background rate | Varies with age | | | N/A | [21] |
| Median age of refugees at arrival | 24 |  |  |  | [22] |
| **Economic parameters** | | | | | |
| *U.S. cost estimates (2013 USD)* | | | | | |
| Screen for all nematodes ^c^ | 116 | 78 | 260 | G | [23-25] |
| Screen for strongyloides, assuming albendazole presumptive treatment ^c^ | 54 | 38 | 113 | G | [23-25] |
| Screen, assuming albendazole + ivermectin presumptive treatment ^c^ | 78 | 57 | 171 | G | [23-25] |
| Albendazole treatment 400mg ^c^ | 173 | 152 | 234 | G | [23-26] |
| Outpatient treatment for intestinal parasites (screening + albendazole cost) ^d^ | 494 | 345 | 1030 | G | [23-26] |
| Outpatient treatment for intestinal parasites (screening + ivermectin cost) ^d^ | 408 | 259 | 940 | G | [23-26] |
| Inpatient treatment for strongyloidiasis ^d^ | 20,000 | 15,000 | 24,000 | G | [27] |
| Ivermectin treatment 18mg ^d^ | 87 | 66 | 150 | G | [23-26] |
| *Overseas cost estimates (2013 USD) ^e^* | | | | | |
| Presumptive albendazole treatment in Asia | 3.2 | 2.7 | 3.7 | G | IOM data |
| Presumptive ivermectin treatment in Asia | 7.6 | 3.8 | 13.3 | G | IOM data |
| *Opportunity cost estimates (2013 USD)* | | | | | |
| Screening (all parasites) ^c^ | 7 | 7 | 22 | G | [28, 29] |
| Screening (*Strongyloides* only) ^c^ | 1 | 1 | 2.20 | G | [28, 29] |
| Treatment after screening ^c^ | 6 | 6 | 22 | G | [28, 29] |
| Outpatient cases ^d^ | 140 | 140 | 176 | G | [28, 29] |
| Inpatient strongyloidiasis ^d^ | 1,400 | 1,400 | 1,760 | G | [27-29] |
| *QALY estimates* | | | | | |
| QALY decrement for *Strongyloides* infections | 0.001 | 0 | 0.01 | B | Assumption |
| QALY decrement for hookworm, *Ascaris*, *Trichuris* infections | 0.001 | 0 | 0.01 | B | Assumption |
| ^a^ Distribution types: U- uniform, B- beta, G- gamma (See Section 5 of appendix for more details)  ^b^ Details in Section 2 of Appendix  ^c^ Details in Section 3 of Appendix  ^d^ Details in Section 4 of Appendix  ^e^ Details in Section 5 of Appendix | | | | | |

1. Swanson SJ, Phares CR, Mamo B, Smith KE, Cetron MS, Stauffer WM. Albendazole therapy and enteric parasites in United States-bound refugees. New England Journal of Medicine. 2012;366(16):1498-507.

2. Caruana SR, Kelly HA, Ngeow JYY, Ryan NJ, Bennett CM, Chea L, et al. Undiagnosed and Potentially Lethal Parasite Infections Among Immigrants and Refugees in Australia. Journal of Travel Medicine. 2006;13(4):233-9.

3. Chang A, Perry S, Du J, Agunbiade A, Polesky A, Parsonnet J. Decreasing intestinal parasites in recent northern California refugees. American Journal of Tropical Medicine and Hygiene. 2013;88(1):191-7.

4. Dawson-Hahn EE, Greenberg SLM, Domachowske JB, Olson BG. Eosinophilia and the Seroprevalence of Schistosomiasis and Strongyloidiasis in Newly Arrived Pediatric Refugees: An Examination of Centers for Disease Control and Prevention Screening Guidelines The Journal of Pediatrics. 2010;156(6):1016-8.e1.

5. Gyorkos TW, Genta RM, Viens P, MacLean JD. Seroepidemiology of Strongyloides infection in the Southeast Asian refugee population in Canada. American Journal of Epidemiology. 1990;132(2):257-64.

6. Paxton G, Sangster K, Maxwell E, CRJ M, Drewe R. Post-arrival health screening in Karen refugees in Australia. PLoS ONE. 2012;7(5):e38194.

7. Krolewiecki AJ, Lammie P, Jacobson J, Gabrielli A-F, Levecke B, Socias E, et al. A Public Health Response against Strongyloides stercoralis : Time to Look at Soil-Transmitted Helminthiasis in Full. PLoS Neglected Tropical Diseases. 2013;7(5):e2165.

8. Keiser J, Utzinger J. Efficacy of Current Drugs Against Soil-Transmitted Helminth Infections Systematic Review and Meta-analysis. Journal of the American Medical Association. 2008;299(16):1937-48.

9. Bisoffi Z, Buonfrate D, Angheben A, Boscolo M, Anselmi M, Marocco S, et al. Randomized Clinical Trial on Ivermectin versus Thiabendazole for the Treatment of Strongyloidiasis. PLoS Neglected Tropical Diseases. 2011;5(7):e1254.

10. Igual-Adell R, Oltra-Alcaraz C, Soler-Company E, Sánchez-Sánchez P, Matogo-Oyana J, Rodríguez-Calabuig D. Efficacy and safety of ivermectin and thiabendazole in the treatment of strongyloidiasis. Expert Opinion on Pharmatherapy. 2004;5(12):2615-9.

11. Suputtamongkol Y, Premasathian N, Bhumimuang K, Waywa D, Nilganuwong S, Karuphong E, et al. Efficacy and Safety of Single and Double Doses of Ivermectin versus 7-Day High Dose Albendazole for Chronic Strongyloidiasis. PLoS Neglected Tropical Diseases. 2011;5(5):e1044.

12. Bisoffi Z, Buonfrate D, Sequi M, Mejia R, Cimino RO, Krolewiecki AJ, et al. Diagnostic Accuracy of Five Serologic Tests for Strongyloides stercoralis Infection. PLoS Neglected Tropical Diseases. 2014;8(1):e2640.

13. Cartwright CP. Utility of Multiple-Stool-Specimen Ova and Parasite Examinations in a High-Prevalence Setting. Journal of Clinical Microbiology. 1999;37(8):2408-11.

14. Nikolay B, Brooker SJ, Pullan RL. Sensitivity of diagnostic tests for human soil-transmitted helminth infections: a meta-analysis in the absence of a true gold standard. International Journal for Parasitology. 2014;44:765-74.

15. Requena-Mendez A, Chiodini P, Bisoffi Z, Buonfrate D, Gotuzzo E, Munoz J. The Laboratory Diagnosis and Follow Up of Strongyloidiasis: A Systematic Review. PLoS Neglected Tropical Diseases. 2013;7(1):e2002.

16. van Doorn HR, Koelewijn R, Hofwegen H, Gilis H, Wetsteyn JCFM, Wismans PJ, et al. Use of Enzyme-Linked Immunosorbent Assay and Dipstick Assay for Detection of Strongyloides stercoralis Infection in Humans. Journal of Clinical Microbiology. 2007;45(2):438-42.

17. Bethony J, Brooker S, Albonico M, Geiger SM, Loukas A, Diemert D, et al. Soil-transmitted helminth infections: ascariasis, trichuriasis, and hookworm. The Lancet. 2006;367:1521-32.

18. Muennig P, Pallin D, Challah C, Khan K. The cost-effectiveness of ivermectin vs. albendazole in the presumptive treatment of strongyloidiasis in immigrants to the United States. Epidemiology and Infection. 2004;132(6):1055-63.

19. Valerio L, Roure S, Fernandez-Rivas G, Basile L, Martınez-Cuevasa O, Ballesteros A-L, et al. Strongyloides stercoralis, the hidden worm. Epidemiological and clinical characteristics of 70 cases diagnosed in the North Metropolitan Area of Barcelona, Spain, 2003–2012. Transactions of the Royal Society of Tropical Medicine and Hygiene. 2013;107:465-70.

20. Muennig P, Pallin D, Sell RL, Chan M-S. The Cost Effectiveness of Strategies for the Treatment of Intestinal Parasites in Immigrants. New England Journal of Medicine. 1999;340(10):773-9.

21. Centers for Disease Control and Prevention, National Center for Health Statistics. Underlying Cause of Death 1999-2010 on CDC WONDER Online Database, released 2012. Data are from the Multiple Cause of Death Files, 1999-2010, as compiled from data provided by the 57 vital statistics jurisdictions through the Vital Statistics Cooperative Program. Accessed at <http://wonder.cdc.gov/ucd-icd10.html> on Jul 8, 2014

22. Department of Homeland Security (2012) Yearbook of Immigration Statistics <http://www.dhs.gov/yearbook-immigration-statistics-2011-0>, accessed on October 1, 2012. Washington, D.C.

23. Centers for Medicare and Medicaid Services. Clinical Laboratory Fee Schedule, <http://www.cms.gov/Medicare/Medicare-Fee-for-Service-Payment/ClinicalLabFeeSched/clinlab.html>. Accessed on March 20, 2013. 2013.

24. Centers for Medicare and Medicaid Services. Physician Fee Schedule, <http://www.cms.gov/Medicare/Medicare-Fee-for-Service-Payment/PhysicianFeeSched/index.html>. accessed on March 20, 2013. 2013.

25. InGauge Healthcare Solutions. 2013 Physicians' Fee & Coding Guide. Atlanta GA2013.

26. Healthcare Series [Red Book]. Greenwood Village, Colo: Thomson Reuters (Healthcare) Inc. Updated periodically. Accessed on May 1, 2013 [Internet]. 2013.

27. HCUP Nationwide Inpatient Sample (NIS). Healthcare Cost and Utilization Project (HCUP). 2007-2009. Agency for Healthcare Research and Quality, Rockville, MD. [www.hcup-us.ahrq.gov/nisoverview.jsp](http://www.hcup-us.ahrq.gov/nisoverview.jsp)

28. Bureau of Labor Statistics, 2013 National Occupational Employment and Wage Estimates. Washington DC, <http://www.bls.gov/oes/current/oes_nat.htm#29-0000>, Accessed on May 1, 2014.

29. 2013 World Economic Outlook database. <http://www.imf.org/external/pubs/ft/weo/2013/01/weodata/index.aspx> Accessed on June 3, 2013. [Internet]. 2013.
