# Supplementary material for: Economic Analysis of the Impact of Overseas and Domestic Treatment and Screening Options for Intestinal Helminth Infection among US-Bound Refugees from Asia
Source: PLoS Negl Trop Dis. 2016 Aug 10;10(8):e0004910. doi: 10.1371/journal.pntd.0004910 (PMC4980012; doi:10.1371/journal.pntd.0004910)
Supplement: S2 Table — (DOCX) [file pntd.0004910.s003.docx]

Table S2. Estimated means and standard deviations for each uncertain parameter included in the analysis

| Description | Base case analysis | Lower bound | Upper bound | Distri-bution^a^ | Mean | Standard Deviation |
| --- | --- | --- | --- | --- | --- | --- |
| ***Epidemiological parameters*** | | | | | | |
| Baseline infection prevalence (without treatment) | | | | | | |
| Hookworm | 0.028 | 0.028 | 0.11 | B | 0.0690 | 0.0006 |
| *Ascaris* | 0.0084 | 0.0084 | 0.017 | B | 0.0127 | 0.0000 |
| *Trichuris* | 0.0056 | 0.0056 | 0.015 | B | 0.0103 | 0.0000 |
| *Strongyloides* | 0.2 | 0.06 | 0.34 | B | 0.2000 | 0.0467 |
| *Drug efficacy* | | | | | | |
| Albendazole against hookworm | 0.72 | 0.72 | 0.93 | B | 0.7550 | 0.0350 |
| Albendazole against *Trichuris* | 0.28 | 0.28 | 0.73 | B | 0.3550 | 0.0750 |
| Albendazole against *Ascaris* | 0.88 | 0.88 | 0.94 | B | 0.8900 | 0.0100 |
| Ivermectin against *Strongyloides* | 0.9 | 0.57 | 0.99 | B | 0.8600 | 0.0700 |
| *Test sensitivity* | | | | | | |
| Sensitivity of *Strongyloides* serologic test | 0.91 | 0.89 | 0.92 | B | 0.9083 | 0.0050 |
| Sensitivity of two stool O&P for hookworm | 0.78 | 0.53 | 0.88 | B | 0.8950 | 0.0150 |
| Sensitivity of two stool O&P for *Ascaris* | 0.81 | 0.57 | 0.95 | B | 0.9583 | 0.0083 |
| Sensitivity of two stool O&P for *Trichuris* | 0.96 | 0.81 | 0.99 | B | 0.9917 | 0.0017 |
| *Test specificity* | | | | | | |
| *Strongyloides* serologic test | 0.92 | 0.89 | 0.97 | B | 0.9233 | 0.0133 |
| Two stool O&P for hookworm*,* *Ascaris*, and *Trichuris* | 1 | 1 | 1 | NA |  |  |
| *Duration of risk of infection for outpatient illness* | | | | | | |
| hookworm | 6 | 5 | 7 | G | 6.0000 | 0.3333 |
| *Trichuris* | 2 | 1 | 2 | G | 1.8333 | 0.1667 |
| *Ascaris* | 1 | 1 | 1 | N/A |  |  |
| *Annual probability of seeking treatment given infection* | | | | | | |
| Outpatient visit for hookworm/ trichuriasis/ascariasis | 0.001 | 0.00012 | 0.005 | B | 0.0015 | 0.0008 |
| Outpatient visit for strongyloidiasis | 0.001 | 0.00012 | 0.005 | B | 0.0015 | 0.0008 |
| Inpatient strongyloidiasis | 2.90E-05 | 6.60E-06 | 1.20E-04 | B | 0.0000 | 0.0000 |
| Case fatality rate for inpatient strongyloidiasis | 0.167 | 0.02 | 0.25 | B | 0.1563 | 0.0383 |
| *Program parameters* | | | | | | |
| Proportion of refugees that present for comprehensive exam in United States | 0.9 | 0.8 | 1 | U | 0.9000 | 0.0033 |
| Probability that refugees will arrive from IOM facilities | 1 | 0.75 | 1 | U | 0.8500 | 0.0033 |
| Probability that refugee will receive presumptive treatment | 0.9 | 0.8 | 0.98 | U | 0.8900 | 0.0027 |
| Probability that stool O&P will be ordered given presumptive treatment | 0.05 | 0.03 | 0.07 | U | 0.0500 | 0.0001 |
| Adjustment factor for overseas versus domestic treatment 0-1 | 1 | 0.75 | 1 | U | 0.9583 | 0.0417 |
| *U.S. cost estimates (2013 USD)* | | | | | | |
| Screen for all nematodes | 116 | 78 | 260 | G | 133.7 | 30.3 |
| Screen for strongyloides, assuming albendazole presumptive treatment | 54 | 38 | 112 | G | 61.0 | 12.3 |
| Screen, assuming albendazole + ivermectin presumptive treatment | 78 | 57 | 171 | G | 90.0 | 19.0 |
| Albendazole treatment 400mg | 173 | 152 | 252 | G | 182.7 | 16.7 |
| Ivermectin treatment 18mg | 87 | 66 | 166 | G | 96.7 | 16.7 |
| Outpatient treatment for intestinal parasites (screening + albendazole cost) | 494 | 345 | 1030 | G | 553.8 | 114.2 |
| Outpatient treatment for intestinal parasites (screening + ivermectin cost) | 408 | 259 | 940 | G | 467.3 | 113.3 |
| Inpatient treatment for strongyloides | 20,000 | 15,000 | 24,000 | G | 19833.3 | 1500.0 |
| *Overseas cost estimates (2013 USD) of presumptive treatment in Asia* | | | | | | |
| Albendazole | 3.2 | 2.7 | 3.7 | G | 3.95 | 0.45 |
| Ivermectin | 7.6 | 3.8 | 13.3 | G | 8.85 | 1.58 |
| *Opportunity cost estimates (2013 USD)* | | | | | | |
| Screening (all parasites) | 7 | 7 | 22 | G | 9.5 | 2.5 |
| Screening (*Strongyloides* only) | 1 | 1 | 2.2 | G | 1.2 | 0.2 |
| Treatment after screening | 6 | 6 | 22 | G | 8.67 | 2.67 |
| Outpatient cases | 140 | 140 | 176 | G | 146 | 6 |
| Inpatient strongyloides | 1,400 | 1,400 | 1,760 | G | 1460 | 60 |
| *QALY estimates* | | | | | | |
| QALY decrement for *Strongyloides* infections | 0.001 | 0 | 0.01 | B | 0.0023 | 0.0017 |
| QALY decrement for hookworm, *Ascaris*, *Trichuris* infections | 0.001 | 0 | 0.01 | B | 0.0023 | 0.0017 |
| ^a^ Distribution types: U- uniform, B- beta, G- gamma | | | | | | |
